# Joint embedding–classifier learning for interpretable collaborative filtering

**DOI:** 10.1186/s12859-024-06026-8

**Published:** 2025-01-22

**Authors:** Clémence Réda, Jill-Jênn Vie, Olaf Wolkenhauer

**Affiliations:** 1 Institute of Computer Science, University of Rostock, 18051 Rostock, Germany; 2 Soda, Inria Saclay, 91120 Palaiseau, France; 3 Leibniz-Institute for Food Systems Biology, 85354 Freising, Germany; 4 Stellenbosch Institute of Advanced Study, Wallenberg Research Centre, Stellenbosch, 7602 South Africa

**Keywords:** Drug repurposing, Interpretability, Gene expression, Collaborative filtering

## Abstract

**Background:**

Interpretability is a topical question in recommender systems, especially in healthcare applications. An interpretable classifier quantifies the importance of each input feature for the predicted item-user association in a non-ambiguous fashion.

**Results:**

We introduce the novel Joint Embedding Learning-classifier for improved Interpretability (JELI). By combining the training of a structured collaborative-filtering classifier and an embedding learning task, JELI predicts new user-item associations based on jointly learned item and user embeddings while providing feature-wise importance scores. Therefore, JELI flexibly allows the introduction of priors on the connections between users, items, and features. In particular, JELI simultaneously (a) learns feature, item, and user embeddings; (b) predicts new item-user associations; (c) provides importance scores for each feature. Moreover, JELI instantiates a generic approach to training recommender systems by encoding generic graph-regularization constraints.

**Conclusions:**

First, we show that the joint training approach yields a gain in the predictive power of the downstream classifier. Second, JELI can recover feature-association dependencies. Finally, JELI induces a restriction in the number of parameters compared to baselines in synthetic and drug-repurposing data sets.

## Background

The Netflix Challenge [1] popularized collaborative filtering, where connections between items and users are inferred based on the guilt-by-association principle and similarities. This approach is particularly suitable for use cases where information about known user-item associations is sparse—typically, close to 99% of all possible user-item associations are not labelled, such as in the MovieLens movie recommendation data set [2]—and when there is implicit feedback. For instance, in the case of movie recommendations on streaming platforms or online advertising, the algorithm often gets only access to clicks, that is, positive feedback. However, the reasons for ignoring an item can be numerous: either the item would straightforwardly receive negative feedback, or the item is too far from the user’s usual exploration zone but could still be enjoyed. In some rare cases, true negative feedback might be accessible but in even smaller numbers than the positive associations, for instance, for drug repurposing data sets, by reporting failed Phase III clinical trials [3]. Collaborative filtering algorithms then enable the modeling of the user’s behavior based on their similarity to other users and the similarity of the potential recommended item to other items positively graded by this cluster of users.

Several types of algorithms implement collaborative filtering. For instance, matrix factorizations [4, 5] such as Non-negative Matrix Factorization (NMF) [6] or Singular Value Decomposition (SVD) [7], decompose the matrix of item-user associations into a product of two low-rank tensors. Other types of algorithms are (deep) neural networks [8–10], which build item and user embeddings with convolutional or graph neural networks based on common associations and/or additional feature values. On the one hand, among those last approaches, graph-based methods, which integrate and infer edges between features, items, and users, seem promising in performance [11]. Predictions are supported by establishing complex connections between those entities. Conversely, matrix factorizations incorporate explicit interpretability, as one can try to connect the inferred latent factors to specific user and item features. One example is the factorization machine (FM) [12], which combines a linear regression-like term and a feature pairwise interaction term to output a score for binary classification. The learned coefficients of the FM explicitly contribute to the score for each item and user feature set. This type of interpretability, called feature attribution in the literature [13–16], allows further downstream statistical analysis of the feature interactions. For instance, in our motivating example of drug repurposing, the objective is to identify novel drug-disease therapeutic associations. If features are genes mutated by the pathology or targeted by the chemical compound, the overrepresented biological pathways among those that are respectively affected or repaired can be retrieved based on the set of key repurposing genes. This, in turn, offers important points to argue in favor of the therapeutic value of a drug-disease indication and for further development towards marketing.

In this work, we aim to combine the performance and versatility (in terms of embeddings) of graph-based collaborative filtering and the explicit interpretability of factorization machines to derive a “best-of-both-worlds” approach for predicting user-item associations. To achieve this, we introduce a special class of factorization machines that leverages a strong hypothesis on the structure of item and user embeddings depending on feature embeddings. This classifier is then jointly trained with a knowledge graph completion task. This knowledge graph connects items, users, and features based on the similarity between them and users and potentially additional priors on their relationships with features. The embeddings used to compute the edge probability scores in the knowledge graph are shared with the factorization machine, which allows the distillation of generic priors into the classifier.

Our paper is structured as follows. In Sect. "Related work", we introduce and give an overview of the state-of-the-art on factorization machines and knowledge graphs and how their combination might be able to overcome some topical questions in the field. Section "Methods" introduces the JELI algorithm, which features our novel class of structured factorization machines and a joint training strategy with a knowledge graph. Eventually, Sect. "Results" shows the performance and interpretability of the JELI approach on both synthetic data sets and drug repurposing applications.

***Notation*** For any matrix *M* (in capital letters), we denote $M_{i,:}$, $M_{:,j}$ and $M_{i,j}$ respectively its $i^\text {th}$ row, $j^\text {th}$ column and coefficient at position (*i*, *j*). For any vector $\varvec{v}$ (in bold type), $\varvec{v}_i$ is its $i^\text {th}$ coefficient. Moreover, $M^{\dagger }$ is the pseudo-inverse of matrix *M*.

## Related work

Our approach, JELI, leverages a generic knowledge graph completion task and the interpretability of factorization machines to derive a novel, explainable collaborative filtering approach.

### Knowledge graph embedding learning

A knowledge graph is a set of triplets of the form (*h*, *r*, *t*) such that the *head* entity *h* is linked to the *tail* entity *t* by the relation *r* [17]. Entity and relation embeddings learned on the graph allow us to capture the structure and connections in the graph in a numerical form, as embeddings are parameters of a function predicting the presence of a triplet in the graph. Those parameters are then learned based on the current set of edges in the graph. This approach encodes the graph structure into numerical representations, which can later be provided to a downstream regression model [18]. The edge prediction function is usually called the interaction model. Many exist [19–22], among these, the Multi-Relational Euclidean (MuRE) model [23], defined for any triplet (*h*, *r*, *t*) of respective embeddings $\varvec{e}^h, \varvec{e}^r, \varvec{e}^t$ of dimension *d* as

1 $$\begin{aligned} \text {MuRE}(\varvec{e}^h, \varvec{e}^r, \varvec{e}^t) = -\Vert R^r \varvec{e}^h - (\varvec{e}^t + \varvec{e}^r)\Vert ^2_2 + b^h + b^t \;, \end{aligned}$$

where $d \times d$ matrix $R^r$, and scalars $b^h$ and $b^t$ are respectively relation-, head- and tail-specific parameters. Notably, this interaction model has exhibited good embedding engineering properties throughout the literature [24, 25].

Yet, many challenges are present in this field of research. Current representation learning algorithms (no matter the selected interaction model between a triplet and its embedding) infer representations directly on the nodes and relations of the graph. However, this approach does not make it possible to establish a relationship between the nodes other than a similarity at the level of the numerical representation for neighboring nodes for specific relations in the graph. That is, specific logical operations depending on the relation are often ignored: for instance, for a relation *r* and its opposite $\lnot r$, we would like to ensure that the score *p* assigned to triplet (*h*, *r*, *t*) is proportional to $-\overline{p}$, where $\overline{p}$ is the score associated with triplet $(h, \lnot r, t)$. Moreover, knowledge graphs are currently more suited to categorical information, where entities and relationships take discrete rather than numerical values. Numerical values could describe a relation such as “users from this specific age group are twice more interested in that movie genre”. Some recent works focus on integrating numerical values into knowledge graph embeddings. In KEN embeddings [26], a single-layer neural network is trained for each numeric relation, taking the attribute as input and returning an embedding. Another approach, TransEA [27], aims to optimize a loss function that linearly combines, with a hyperparameter, a loss value on the categorical variables (the difference between the scores and the indicator of the presence of a triplet) and another loss value on numerical variables, which seeks to minimize the gap between the variable and a scalar product involving its embedding. However, these two approaches add several additional hyperparameters and do not deal with interpretability.

Resorting to knowledge-graph-infused embeddings allows us to integrate prior knowledge constraints generically into the representations of entities, both items and users. We aim to enforce a structure on those embeddings to guarantee the good prediction of user-item associations by incorporating those embeddings into a special type of factorization machine.

### Factorization machines

Factorization machines are a type of collaborative filtering algorithms introduced by [12]. Their most common expression, the second-order factorization machine of dimension *d*, comprises a linear regression term of coefficient (with a possibly non-zero intercept) and a term that combines interactions from all distinct pairs of features by featuring a scalar product of their corresponding low-rank latent vectors of dimension *d*. This approach, particularly in the presence of sparse feature vectors, is computationally efficient while performant on a variety of recommendation tasks: for instance, knowledge tracing for education [28], click-through rate prediction [29]. Computationally tractable evaluation and training routines were first proposed by [30] for higher-order factorization machines (HOFMs), which were introduced as well in [12] and include interactions from all distinct *K* sets of features, where $K \ge 2$, opening the way to even finer classification models. The definition of HOFMs is recalled in Definition 1.

#### Definition 1

Higher–Order Factorization Machines (HOFMs). Let us denote the set of available item and user features $\mathcal {F} \subseteq \mathbb {N}^*$. The general expression for HOFM [12, 30] of order $m \ge 2$ and dimensions $d_2,\dots ,d_m$ that takes as input a single feature vector $\varvec{x} \in \mathbb {R}^{|\mathcal {F}|}$ is a model such that $\varvec{\theta } = (\omega ^0,\varvec{\omega }^1,\varvec{\omega }^2,\dots ,\varvec{\omega }^m)$ where $(\omega ^0, \varvec{\omega }^1) \in \mathbb {R} \times \mathbb {R}^{|\mathcal {F}|}$ and for any $i \in \{2,\dots ,m\}$, $\varvec{\omega }^i \in \mathbb {R}^{|\mathcal {F}| \times d_i}$

2 $$\begin{aligned} \text {HOFM}_\theta (\varvec{x}) \triangleq \omega ^0 + (\varvec{\omega }^1)^\intercal \varvec{x} + \sum _{2 \le t \le m} \sum _{\begin{array}{c} f_1< \dots < f_t\\ f_1,\dots ,f_t \in \mathcal {F} \end{array}} \langle \varvec{\omega }^t_{f_1,:}, \dots , \varvec{\omega }^t_{f_t,:} \rangle \varvec{x}_{f_1} \cdot \varvec{x}_{f_2} \cdot \dots \cdot \varvec{x}_{f_{t-1}} \cdot \varvec{x}_{f_t}\;, \end{aligned}$$

where $\langle \varvec{\omega }^t_{f_1,:}, \dots , \varvec{\omega }^t_{f_t,:} \rangle \triangleq \sum _{d \le d_t} \varvec{\omega }^t_{f_1,d} \cdot \varvec{\omega }^t_{f_2,d} \cdot \dots \cdot \varvec{\omega }^t_{f_{t-1},d} \cdot \varvec{\omega }^t_{f_t,d}$ for any *t* and indices $f_1,\dots ,f_t$. In particular, for $m=2$

3 $$\begin{aligned} \text {FM}_\theta (\varvec{x}) \triangleq \overbrace{\omega ^0 + (\varvec{\omega }^1)^\intercal x}^\text {linear regression term} + \overbrace{\sum _{f < f', f,f' \in \mathcal {F}} \langle \varvec{\omega }^2_{f,:},\varvec{\omega }^2_{f',:} \rangle \varvec{x}_{f} \cdot \varvec{x}_{f'}}^\text {pairwise interaction term}\;. \end{aligned}$$

Besides their good predictive power, factorization machines involve explicit coefficients that quantify the contribution of each *K* set of features to the final score associated with the positive class of associations. These coefficients offer a straightforward insight into the discriminating features for the recommendation problem, and this type of “white-box” explainability is related to a larger research field called feature attribution-based interpretability.

### Feature attribution-based interpretability

Given a binary classifier *C* and a feature vector $\varvec{x} \in \mathbb {R}^F$, a feature attribution function $\phi ^C : \mathbb {R}^F \rightarrow \mathbb {R}^F$ returns importance scores for each feature contributing to the positive class score for the input vector $\varvec{x}$. If the importance score associated with feature *f* is largely positive (resp., negative), it means that feature *f* drives the membership of $\varvec{x}$ to the positive (resp., negative) class. In contrast, an importance score close to 0 indicates that feature *f* has little influence on the classification of data point $\varvec{x}$. Albeit other types of interpretability approaches exist (based on decision rules given by single classifier trees or random forests [31, 32], counterfactual examples [33] or logic rules [34, 35]) the importance score-based methods allow going beyond single feature influence. In particular, the importance scores can be integrated into downstream analyses to statistically quantify the effect of specific groups of features on the classification. For instance, when considering genes as features, an enrichment analysis [36] based on the scores can uncover overrepresented functionally consistent cell pathways.

Some classifiers, as seen for factorization machines, readily include importance scores, whereas several approaches compute post-hoc importance scores. Importance scores are evaluated based on the outputs of an already trained “black-box” classifier, such as a neural network. Such approaches include Shapley values [13], LIME [14], DeepLIFT [37] (for image annotation) or sufficient explanations [38]. Yet, recent works show their lack of robustness and consistency across post-hoc feature attribution methods, both empirically [15] and theoretically [16, 39]. However, the advantage of posthoc approaches is that they allow the explainability of any type of classifier and combine the richness of the model (predictive performance) and interpretability.

The approach described in our paper then aims to encompass any generic embedding model without losing the connection to the initial features of the input vectors to the classifier.

## Methods

**Fig. 1:**
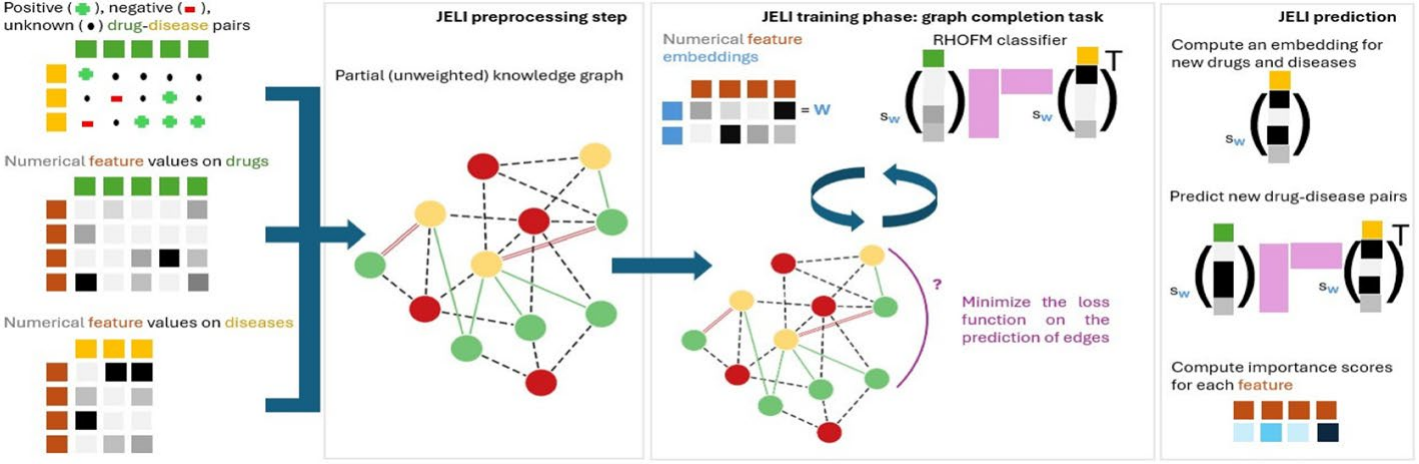
Full pipeline of the JELI algorithm, from the initial inputs to the downstream tasks

In this section, we define the JELI algorithm, our main contribution. The full pipeline of JELI is illustrated in Fig. 1. Let us define in formal terms the inputs to the associated recommendation problem of $n_i$ items $i_1,i_2,\dots ,i_{n_i}$ to $n_u$ users $u_1,u_2,\dots ,u_{n_u}$. The minimal input to the recommendation problem is the user-item association matrix $A \in \{-1,0,+1\}^{n_i \times n_u}$ which summarizes the known positive ($+1$)—and possibly negative ($-1$)—associations and denotes unknown associations by zeroes. In simple terms, the recommender systems aim to replace zeroes by $\pm 1$ while preserving the label of nonzero-valued associations. Second, in some cases, we also have access to the respective item and user feature matrices denoted $S \in \mathbb {R}^{F \times n_i}$ and $P \in \mathbb {R}^{F \times n_u}$. Without a loss of generality, we assume that the item and user feature matrices have the same *F* features $f_1, f_2, \dots , f_{F}$. 1 Finally, there might be a partial graph on some of the items, users, features, and possibly other entities. For instance, such a graph might connect movies, users, and human emotions for movie recommendation [40], or drugs, diseases, pathways, and proteins or genes for drug repurposing [41, 42]. We denote this graph $\mathcal {G}(\mathcal {V}_\mathcal {G}, \mathcal {E}_\mathcal {G})$, where $\mathcal {V}_\mathcal {G}$ is the set of nodes in $\mathcal {G}$ and $\mathcal {E}_\mathcal {G}$ is its set of (undirected, labeled) edges.

We first introduce the class of higher-order factorization machines, called redundant structured HOFMs, which will classify user-item associations based on an assumption on the structure of item/user and feature embeddings.

### Redundant structured HOFM (RHOFM)

This subtype of higher-order factorization machines features shared higher-order parameters across interaction orders, such that the corresponding dimensions of the HOFM satisfy $d_2=\dots =d_m=d$ in Definition 1. As such, RHOFMs are related to inhomogeneous ANOVA kernel HOFMs (iHOFMs) mentioned in [30]. This type of factorization machine is such that the higher-order dimensions are all equal (that is, $d_2=\dots =d_m=d$) and the corresponding higher-order coefficients are all proportional to one another: for any $t,t' \ge 2$ and $f \le F$. there exists $c \in \mathbb {R}$ such that $\varvec{\omega }^t_f = c \cdot \varvec{\omega }^{t'}_f$ in Definition 1. However, what distinguishes the RHOFM from an iHOFM is the following hypothesis on structure: it is assumed that every entity *d*-dimensional embedding $\varvec{e} \in \mathbb {R}^d$ results from some function $s_W$ with parameter $W \in \mathbb {R}^{F \times d}$ applied to the corresponding entity feature vector $\varvec{x} \in \mathbb {R}^F$. For instance, an embedding $\varvec{e}$ associated with feature vector $\varvec{x}$ with a *linear structure function of dimension d* is defined as $\varvec{e} = s_W(\varvec{x}) = \varvec{x} W$. However, any, possibly non-linear, structure function $s_W$ can be considered. Note that for completeness, we can define a feature vector for *features*, which is simply the result of the indicator function on features in *F*: for feature $f \in F$, its corresponding feature vector is $\varvec{x}^f \triangleq (\delta _{(f_j=f)})_{j \le F}$ where $\delta$ is the Kronecker symbol, such that the structure function $s_W$ can be applied to any item, user or feature entity. Definition 2 gives the formal expression of RHOFMs for any order, dimension, and structure.

#### Definition 2

Redundant structured HOFMs (RHOFMs). The RHOFM of structure $s_W$, order *m* and dimension *d*, with parameters $\varvec{\theta } = (\omega ^0, \varvec{\omega }^1, \varvec{\omega }^{2:m}, W) \in \mathbb {R} \times \mathbb {R}^{d} \times \mathbb {R}^{m-1} \times \mathbb {R}^{F \times d}$ on item and user of respective feature vectors $\varvec{x}^i, \varvec{x}^u \in \mathbb {R}^F$ is defined as

4 $$\begin{aligned} \text {RHOFM}_{\varvec{\theta }}\,(\varvec{x}^i, \varvec{x}^u)\triangleq & \omega ^0 + (\varvec{\omega }^1)^\intercal (\varvec{x}'^{iu})^\intercal \begin{bmatrix}\widetilde{W}^{iu}_\lambda \\ \widetilde{W}^{iu}_\lambda \end{bmatrix}\\+ & \sum _{2 \le t \le m} \varvec{\omega }^{2:m}_{t-1} \sum _{\begin{array}{c} f_1< \dots < f_t\\ f_1,\dots ,f_t \le 2F \end{array}} \left\langle \begin{bmatrix}\widetilde{W}^{iu}_\lambda \\ \widetilde{W}^{iu}_\lambda \end{bmatrix}_{f_1,:},\ldots , \begin{bmatrix}\widetilde{W}^{iu}_\lambda \\ \widetilde{W}^{iu}_\lambda \end{bmatrix}_{f_t,:} \right\rangle \varvec{x}'^{iu}_{f_1}\varvec{x}'^{iu}_{f_2} ... \varvec{x}'^{iu}_{f_t}\;, \nonumber \end{aligned}$$

where $\varvec{x}'^{iu} \triangleq [(\varvec{x}^i)^\intercal , (\varvec{x}^u)^\intercal ]^\intercal \in \mathbb {R}^{2F}$ is the concatenation of feature vectors along the row dimension, $\widetilde{\varvec{x}}^{iu} \triangleq [\varvec{x}^i, \varvec{x}^u]^\intercal \in \mathbb {R}^{F \times 2}$ the concatenation along the column dimension, $\widetilde{W}^{iu}_\lambda \triangleq (\widetilde{\varvec{x}}^{iu} (\widetilde{\varvec{x}}^{iu})^\intercal + \lambda I_F)^{\dagger }(\widetilde{\varvec{x}}^{iu})^\intercal [s_W(\varvec{x}^i)^\intercal , s_W(\varvec{x}^u)^\intercal ] \in \mathbb {R}^{F \times d}$ is the $\lambda$-regularized approximate least squares estimator in the following equation in *V*: $s_W(\widetilde{\varvec{x}}^{iu}) = \widetilde{\varvec{x}}^{iu} V$, with $\lambda \ge 0$.

By reordering terms and by definition of $\widetilde{W}^{iu}_\lambda$ (full details in Appendix ), if we denote $f \% F$ the remainder of the Euclidean division of *f* by *F*, we can notice that

5 $$\begin{aligned} \text {RHOFM}_{\varvec{\theta }}(\varvec{x}^i, \varvec{x}^u)\approx & \omega ^0 + (\varvec{\omega }^1)^\intercal (s_W(\varvec{x}^i)+s_W(\varvec{x}^u))\\+ & \sum _{2 \le t \le m} \varvec{\omega }^{2:m}_{t-1} \sum _{\begin{array}{c} f_1< \dots < f_t\\ f_1,\dots ,f_t \le 2F \end{array}} \left\langle \varvec{x}'^{iu}_{f_1} s_W(\varvec{x}^{f_1\%F}),\ldots , \varvec{x}'^{iu}_{f_t} s_W(\varvec{x}^{f_t\%F}) \right\rangle \;.\nonumber \end{aligned}$$

In particular, for $m=2$, $\text {RHOFM}_{\varvec{\theta }}(\varvec{x}^i, \varvec{x}^u)$ is roughly 2 equal to

6 $$\begin{aligned} & \omega ^0 + (\varvec{\omega }^1)^\intercal \Big (s_W(\varvec{x}^i)+s_W(\varvec{x}^u)\Big ) + \omega ^{2} \sum _{\begin{array}{c} f_1 < f_2\\ f_1,f_2 \le 2F \end{array}} \left\langle \varvec{x}'^{iu}_{f_1} s_W(\varvec{x}^{f_1\%F}), \varvec{x}'^{iu}_{f_2} s_W(\varvec{x}^{f_2\%F}) \right\rangle \;. \qquad \end{aligned}$$

Compared to the expression of a factorization machine for $m=2$ in Eq. (3), the RHOFM includes a structure that can be non-linear (through the function $s_W$) and a supplementary degree of freedom with parameters $\varvec{\omega }^{1}$ and $\varvec{\omega }^{2:m}$.

The RHOFM then comprises a term linear in the item/user embeddings and a product of feature embeddings weighted by the corresponding values in the item and user initial feature vectors. Moreover, if we assume a linear structure on the RHOFM, the embedding vector for feature $f_j$ is exactly $W_{f_j,:}$ and the embeddings for items and users are the sum of feature embeddings weighted by their corresponding values in the item and user vectors. The expression in Definition 2 is relatively computationally efficient when combined with the dynamic programming routines described in [30]. Moreover, the redundancy in the RHOFM allows it to benefit from the same type of computational speed-up as inhomogeneous ANOVA kernels or iHOFMs.

Knowing that HOFMs (in Definition 1) and iHOFMs would take as input the concatenation along the row dimension of $(\varvec{x}^i, \varvec{x}^u)$, assuming that the dimensions across subsets are the same, *i*.*e*., $d_2=\dots =d_m=d$, HOFMs comprise $1+2F+2Fd(m-1)$ parameters, which can account for a prohibitive computation cost in practice. Similarly, iHOFMs would require the training of $1+m+2Fd$ parameters, whereas RHOFMs (in Definition 2) only feature $1+m+(F+1)d$, hence removing the multiplicative constant on the number of features *F*, which has an impact for high-dimensional data sets such as the TRANSCRIPT drug repurposing data set [43] which gathers values on 12, 000 genes across the human genome.

Regarding interpretability, as evidenced by Eq. (5), the coefficients involved in the expression of the RHOFM are straightforwardly connected to the input embeddings. In the case of the linear structure and when $\varvec{\omega }^1=\varvec{1}_d$, $\varvec{\omega }^{2:m}=\varvec{1}_{m-1}$ (or any other constant), the contributions from features on the one hand and the item/user values on the other can easily be disentangled. In that case, $\widetilde{W}^{iu}_\lambda \approx W$ and then for any feature *f*, the intrinsic (*i*.*e*., independent of users or items) importance score is $\sum _{k \le d} \ W_{f,k}$. When associated with an entity (item or user) of feature vector $\varvec{x} \in \mathbb {R}^F$, its importance score is simply $\varvec{x}_f \sum _{k \le d} \ W_{f,k}$. Using $\widetilde{\varvec{x}}^{iu}\widetilde{W}^{iu}_\lambda \approx s_W(\widetilde{\varvec{x}}^{iu})$ in non-linear structures, we can extrapolate this result to obtain the following intrinsic feature importance score

#### Result 1

Feature importance scores in a RHOFM. When $\varvec{\omega }^1=\varvec{1}_d$, $\varvec{\omega }^{2:m}=\varvec{1}_{m-1}$ (or any other constant), the intrinsic (entity-independent) feature importance score for feature $f \le F$ in an RHOFM (Definition 2) is $\sum _{k \le d} \ (\widetilde{W}^{iu}_\lambda )_{f,k} \;.$ As a consequence, the feature attribution function associated with feature vector $\varvec{x} \in \mathbb {R}^F$ is $\phi ^\text {RHOFM}(\varvec{x}) \triangleq (\varvec{x}_f\sum _{k \le d} (\widetilde{W}^{iu}_\lambda )_{f,k})_{f\le F} \,.$

One could infer the RHOFM parameters by directly minimizing a loss function. However, as mentioned in the introduction, we would like to distil some prior knowledge information into the RHOFM, for instance, via a knowledge graph specific to the recommendation use case. By seeing the feature embeddings in the RHOFM as node embeddings in a knowledge graph, the next section describes how to jointly train the RHOFM and the feature embeddings on a knowledge graph completion task.

### Joint training of the RHOFM and the knowledge graph embeddings

We will leverage the information from the partial graph $\mathcal {G}(\mathcal {V}_\mathcal {G}, \mathcal {E}_\mathcal {G})$ to fit the RHOFM, by reducing the problem of classification to the prediction of a subset of edges in a knowledge graph completion problem. To do so, we first extend the partial graph $\mathcal {G}$ based on the respective user-item association matrix *A*, and respective item and user feature matrices *S* and *P* to build a knowledge graph $\mathcal {K}(\mathcal {V}, \mathcal {T})$ with nine types of relations. Note that the partial graph can possibly be empty or, to the contrary, can include any edge between drugs and features, diseases and features, and between two features.

#### Definition 3

Similarity-based knowledge graph augmented with prior edges. Considering a similarity threshold $\tau \in [0,1]$ associated with a similarity function sim $: \mathbb {R}^F \times \mathbb {R}^F \rightarrow [-1,1]$, JELI builds a knowledge graph from the data set *A*, *P* and *S* and partial graph $\mathcal {G}(\mathcal {V}_\mathcal {G}, \mathcal {E}_\mathcal {G})$ as follows

7 $$\begin{aligned} \mathcal {V}\triangleq & \{i_1,i_2,\dots ,i_{n_i}\} \cup \{u_1,u_2,\dots ,u_{n_u}\} \cup \{f_1,f_2,\dots ,f_{F}\}\;, \end{aligned}$$

8 $$\begin{aligned} \mathcal {T}\triangleq & \{ (s, \text {prior}, t) \mid (s,t) \in \mathcal {E}_\mathcal {G}, s, t \in \mathcal {V} \} \\&\cup\{ (i_j, -, u_k) \mid A_{i_j,u_k}=-1, j \le n_i, k \le n_u \}\nonumber \\&\cup \{ (i_j, +, u_k) \mid A_{i_j,u_k}=+1, \ j \le n_i, \ k \le n_u \} \nonumber \\&\cup \{ (u_j, \text {user-sim}, u_k) \mid \texttt {sim}(P_{:,u_j}, P_{:,u_k})> \tau , \ j,k \le n_u \}\nonumber \\&\cup \{ (i_j, \text {item-sim}, i_k) \mid \texttt {sim}(S_{:,i_j}, S_{:,i_k})> \tau , \ j,k \le n_i \} \nonumber \\&\cup \{ (i_j, \text {item-feat-pos}, f_k) \mid S_{f_k,i_j}> 0, \ k \le F, \ j \le n_i \}\nonumber \\&\cup \{ (i_j, \text {item-feat-neg}, f_k) \mid S_{f_k,i_j}< 0, \ k \le F, \ j \le n_i \} \nonumber \\&\cup \{ (u_j, \text {user-feat-pos}, f_k) \mid P_{f_k,u_j}> 0, \ k \le F, \ j \le n_u \}\nonumber \\&\cup \{ (u_j, \text {user-feat-neg}, f_k) \mid P_{f_k,u_j} < 0, \ k \le F, \ j \le n_u \}\;. \nonumber \end{aligned}$$

The objective of knowledge graph completion is to fit a model predictive of the probability of the presence of a triplet in the knowledge graph. In particular, computing the score associated with triplets of the form $(h, +, t)$, for (*h*, *t*) a user-item pair, boils down to fitting a classifier of user-item interactions. Conversely, a straightforward assumption is that the score associated with triplets $(h, +, t)$ should be opposite to the score assigned to triplets $(h, -, t)$. With that in mind, denoting the set of RHOFM parameters $\varvec{\theta }$ and $\varvec{\theta }^\text {JELI} \triangleq (\varvec{\theta }, \{ R_r, \ r \text { relation} \}, \{ \varvec{e}_r, \ r \text { relation} \}, \{b_h, \ h \in \mathcal {V}\})$ as the total set of parameters to estimate, we define in Eq. (9) the edge score to be maximized for present triplets in the knowledge graph $\mathcal {K}$

9 $$\begin{aligned} \text {score}_{\varvec{\theta }^\text {JELI}}(h, r, t) \triangleq {\left\{ \begin{array}{ll} \ \text {MuRE}(s_W(\varvec{x}^h), \varvec{e}_r, s_W(\varvec{x}^t); R_r, b_h, b_t) & \text { if } r \not \in \{+,-\}\\ \ \text {RHOFM}_{\varvec{\theta }}(\varvec{x}^h, \varvec{x}^t) & \text { if } r=+ \\ \ -\text {RHOFM}_{\varvec{\theta }}(\varvec{x}^h, \varvec{x}^t) & \text { if } r=- \end{array}\right. } \;. \end{aligned}$$

Remember that the vector $\varvec{x}^h$ is well-defined for any item, user, or feature *h*. Then we fit parameter $\varvec{\theta }^\text {JELI}$ by minimizing the soft margin ranking loss with margin $\lambda ^0 = 1$, which expression is recalled below

10 $$\begin{aligned} \forall \varvec{\theta }' \ , \ \text {L}^\text {margin}(\varvec{\theta }') \triangleq \sum _{(h, r, t) \in \mathcal {T}} \sum _{(\overline{h}, r, t) \notin \mathcal {T}} \log \Big ( 1 + \exp \big (\lambda ^0 + \text {score}_{\varvec{\theta }'}(h, r, t)-\text {score}_{\varvec{\theta }'}(\overline{h}, r, t)\big ) \Big ) \,. \end{aligned}$$

Further implementation details and numerical considerations for the training pipeline are available in Appendix .

### Downstream tasks with JELI

Interestingly, not only does JELI build embeddings for items and users available at training time, but it can also be used to produce embeddings for new entities without requiring any retraining step. Given a feature vector $\varvec{x} \in \mathbb {R}^F$, padding with zeroes if needed on unavailable features, the corresponding embedding is $s_W(\varvec{x})$. However, the main objective of the trained JELI model is to predict new (positive) user-item associations, possibly on items and users not observed at training time. In that case, for any pair of item and user feature vectors $(\varvec{x}^i, \varvec{x}^u) \in \mathbb {R}^F \times \mathbb {R}^F$, the label predicted by JELI with RHOFM parameter $\varvec{\theta }$ is

11 $$\begin{aligned} \hat{y}^\text {JELI}(\varvec{x}^i, \varvec{x}^u) \triangleq {\left\{ \begin{array}{ll} \ +1 & \text { if } \sigma (\text {RHOFM}_{\varvec{\theta }}(\varvec{x}^i, \varvec{x}^u))>0.5\\ \ -1 & \text { otherwise} \end{array}\right. } \;, \end{aligned}$$

where $\sigma$ is the standard sigmoid function. Note that the JELI approach could be even more generic. Besides any knowledge graph, this joint training approach could feature any classifier, and not necessarily an RHOFM, as long as the classifier remains interpretable, and any knowledge graph completion loss function or any edge score function.

## Results

We first validate the performance, the interpretability, and the different components of JELI on synthetic data sets, for which the ground truth on feature importance is available. Then, we apply JELI to drug repurposing, our main motivating example for interpretability in recommendation. Further information about the generation of the synthetic data sets and numerical details is available in Appendix . Unless otherwise specified, the order of all factorization machine variants considered (including the RHOFM classifier in JELI) satisfies $m=2$.

**Table 1:** Description of the performance metrics in Section "Results"

Notation	Performance metric	Definition
Spearman’s $\rho$	Spearman’s correlation	$1-6 \sum _{f \le F} (\Delta _f)^2 /(F(F^2-1))$
AUC	Area Under the Curve	$\int _0^1 \text {TPR}(\text {FPR}^{-1}(\tau ; \hat{A}, A) ; \hat{A}, A)d\tau$
NS-AUC	Average NS-AUC [44]	$|n_u|^{-1} \sum _{u \le n_u} |\widetilde{\Omega }_u|^{-1} \sum _{(i,i') \in \widetilde{\Omega }_u} \delta (\hat{A}_{i,u}>\hat{A}_{i',u})$
NDCG	Average NDCG@$n_i$	$n_u^{-1} \sum _{u \le n_u} \left( \sum _{i=1}^{N^+_u} \frac{A_{\sigma _{u}(i), u}}{\log _2(i+1)} \right) /\left( \sum _{i=1}^{N^+_u} \frac{1}{\log _2(i+1)} \right)$

Spearman’s $\rho$: $\Delta _f$ is the gap in rank (for the decreasing order) between the true and predicted importance scores $(s^\star )_f$ and $\hat{s}_f$ for feature *f*. AUC: The true positive rate between ground truth *A* and predictions $\hat{A}$ is defined as $\text {TPR}(\tau ; \hat{A}, A) = \sum _{(i,u),A_{i,u}=+1} \delta (\hat{A}_{i,u}>\tau )/\sum _{(i,u)} \delta (\hat{A}_{i,u}>\tau )$, the false positive rate is $\text {FPR}(\tau ; \hat{A}, A) = \sum _{(i,u),A_{i,u}=-1} \delta (\hat{A}_{i,u}>\tau )/\sum _{(i,u)} \delta (\hat{A}_{i,u}\le \tau )$, and $\delta$ is the Kronecker symbol. NS-AUC: The set of true positive, respectively negative, drug-disease associations is $\Omega ^\pm \triangleq \{(i,u), A_{i,u} = \pm 1 \mid i \le n_i, u \le n_u\}$, whereas the set of positive drugs to disease *u* is $\Omega ^+_u \triangleq \{i \mid A_{i,u} = +1\}$. Finally, the set of correctly ranked drugs for disease *u* is $\widetilde{\Omega }_u \triangleq \{(i,i') \mid A_{i,u}> A_{i',u}\}$. NDCG: $\sigma _{u}$ is the permutation that sorts all coefficients of the recommendations $\hat{A}_{i,u}, i \le n_i$ for disease *u* in the decreasing order. That is, $\hat{A}_{\sigma _u(1),u} \ge \hat{A}_{\sigma _u(2),u} \ge \dots \ge \hat{A}_{\sigma _u(n_i),u}$. Finally, $N^+_u$ is defined as $\min (n_i,|\Omega ^+_u|)$.

In this section, we consider several evaluation metrics. First, Spearman’s rank correlation [45] quantifies the quality of the importance scores. It is computed on ground truth importance scores $\varvec{s}^\star \triangleq (\sum _{k \le d} W^\star _{f,k})_{f \le F}$ and predicted ones $\hat{\varvec{s}} \triangleq (\sum _{k \le d} \widehat{W}_{f,k})_{f \le F}$ with $\widehat{W}$ the inferred embedding parameter. Second, the Area Under the Curve (AUC) is computed on all user-item pairs to measure classification performance between the ground truth $A \in \{-1,0,+1\}^{n_i \times n_u}$ and the classifier scores $\hat{A} \in \mathbb {R}^{n_i \times n_u}$. We also consider the Negative-Sampling AUC (NS-AUC) [44]. Contrary to AUC, the NS-AUC is a ranking measure akin to an average of user-wise AUCs, giving a more refined quantification of prediction quality across users. As a complementary measure of classification quality, we also consider the Normalized Discounted Cumulative Gain (NDCG), which is proportional to the quality of the ranking of recommended drugs across diseases. Note that all those classification metrics depend solely on the classifier scores, and not on the final class labels that can be inferred by applying a fixed threshold $\tau$. The exact definitions of each metric are reported in Table 1.

### Synthetic data sets

We consider two types of “interpretable” synthetic recommendation data, called “linear first-order” and “linear second-order”, for which the ground truth feature importance scores are known. At fixed values of dimension *d*, feature number *F*, and numbers of items and users $n_i$ and $n_u$, both item and user feature vectors are drawn at random from a standard Gaussian distribution, along with a matrix $W^\star \in \mathbb {R}^{F \times d}$. The algorithm cannot access the full feature values in most practical cases in recommendation tasks. Reasons for missing values can be diverse [46], but most likely follow a *not missing at random* mechanism, meaning that the probability of a missing value depends on the features. To implement such a mechanism, we applied a slightly adapted Gaussian self-masking [47] to the corresponding item and user feature matrices, such that we expect around $10\%$ of missing feature values.

The complete set of user-item scores is obtained by a generating model $g_0 : \mathbb {R}^F \times \mathbb {R}^F \rightarrow [0,1]$. For “first-order” synthetic data sets, $g_0$ is defined as $(\varvec{x}^i, \varvec{x}^u) \mapsto \sigma (\sum _{k \le d} (\varvec{x}^i+\varvec{x}^u) W^\star _{:,k}) = \sigma (\text {RHOFM}_{(0,\varvec{1}_d, \varvec{0}_{m-1}, W^\star )}(\varvec{x}^i, \varvec{x}^u))$ where $x^i$ and $x^u$ are respectively the item and user feature vectors. For the “second-order” type, $g_0$ is simply $(\varvec{x}^i, \varvec{x}^u) \mapsto \sigma (\text {RHOFM}_{(1,\varvec{1}_d, \varvec{1}_{m-1}, W^\star )}(\varvec{x}^i, \varvec{x}^u))$ where the order is $m=2$. In both cases, the corresponding structure function $s_{W^\star }$ is linear, that is, $s_{W^\star }(\varvec{x}) = \varvec{x} W^\star$ and $\lambda = 0$.

Finally, since in practice, most of the user-item associations are inaccessible at training time, we label user-item pairs with $-1$, 0, and $+1$ depending on their score, such that the *sparsity number*—that is, the percentage of unknown values in the association matrix—is equal to a prespecified value greater than $50\%$.

#### JELI is performant for various validation metrics and reliably retrieves ground truth importance scores

**Table 2:** Average validation metrics with standard deviations across 100 iterations and 10 synthetic data sets of each type (total number of values: 1000)

Data set type	AUC	NS-AUC	Spearman’s $\rho$
First-order	$0.99 \pm$ 0.013	$0.89 \pm$ 0.124	$0.83 \pm$ 0.279
Second-order	$0.98 \pm$ 0.019	$0.86 \pm$ 0.167	$0.75 \pm$ 0.363

Average (respectively, standard deviation) values are rounded to the closest second (resp., third) decimal place. AUC: Area Under the Curve. NS-AUC: Negative-Sampling AUC [44]. Spearman’s $\rho$: Spearman’s rank correlation

We generate 10 synthetic datasets of each type ($F=10$, $d=2$, $n_i=n_u=173$) and run JELI 100 times with different random seeds corresponding to different training/testing splits. Table 2 shows the numerical results across those $10 \times 100$ runs for several validation metrics on the predicted item-user associations and feature importance scores.

**Fig. 2:**
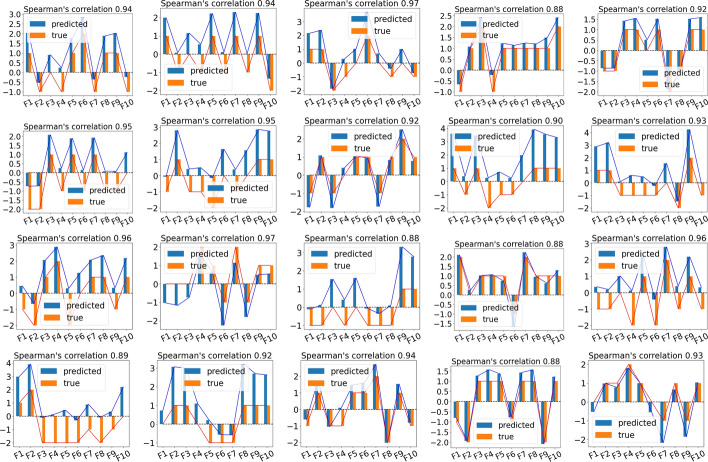
Barplots of the true and predicted feature importance scores for $F=10$ features in each synthetic data set for the best-performing model across 100 iterations. Top-2 lines: on “first-order” synthetic data. Bottom-2 lines: on “second-order” synthetic data

Albeit there is a large variation in the quality of the prediction due to the random training/testing split when considering the average best value across 100 iterations, the metrics in Table 2 show a high predictive power for JELI, along with a consistently high correlation between true and predicted feature importance scores: the average Spearman’s rank correlation for the best-trained models across all 10 data sets is 0.932 for “first-order” sets and 0.932 for “second-order” ones. The bar plots representing the ground truth and predicted importance scores for each of these 10 sets and each type of synthetic data in Fig. 2 show that JELI can preserve the global trend in importance scores across data sets. We also tested the impact of the dimension parameter *d* and of the order *m* of the RHOFM on the accuracy metrics. In the previous experiments, we used $d=2$, which is the true dimensionality of the underlying generating model. However, it appears that JELI is also robust to the choice of the dimension parameter if it is large enough for all metrics. Moreover, similarly to higher-order factorization machines, higher-order interactions ($m>2$) allow us to get a more expressive classifier model and, thus, better classification performance. However, this improvement comes at a heavy computational price, even with the dynamic programming routines in [30], where the time complexity is linear in *m*. The experiments and results on parameter impact can be found in Appendix 4.

#### JELI is robust in synthetic data sets across sparsity numbers

**Fig. 3:**
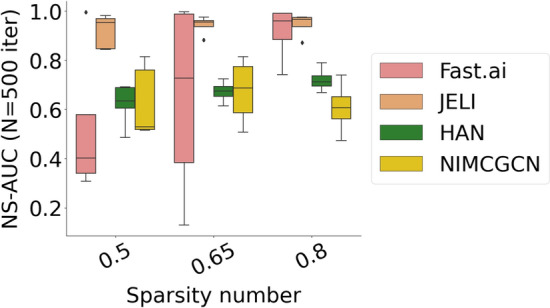
NS-AUC values across “first-order” synthetic data sets for sparsity numbers and 500 iterations for JELI and state-of-the-art embedding-based recommender systems

We also compare the predictive performance of JELI compared to embedding-based recommender systems from the state-of-the-art, namely Fast.ai collaborative learner [8], the heterogeneous attention network (HAN) algorithm [48] and the neural inductive matrix completion with graph convolutional network (NIMCGCN) [10]. We set, whenever appropriate, the same hyperparameter values for all algorithms (with $d=2$). We run each algorithm on 100 different random seeds on 5 “first-order” synthetic data sets generated with sparsity numbers in $\{50\%, 65\%, 80\%\}$, for 500 tests. Figure 3 and Table 3 report the boxplots and the confidence intervals on corresponding validation metrics. In addition to the AUC and NS-AUC, we include the Non-Discounted Cumulative Gain (NDCG) computed for each user at rank $n_i$ (number of items) and averaged across users as a counterpart to the NS-AUC measure.

As illustrated by Fig. 3, JELI consistently outperforms the state-of-the-art on all metrics and remains robust to the sparsity number.

**Table 3:** Average metrics with standard deviations across 100 iterations and 5 “first-order” sets

		AUC	NS-AUC	NDCG
50%	Fast.ai	**0.99± 0.0**	0.52± 0.3	0.85± 0.1
	HAN	0.93± 0.0	0.62± 0.1	0.18± 0.1
	NIM	0.93± 0.0	0.63± 0.1	0.39± 0.1
	JELI	**0.99± 0.0**	**0.92± 0.1**	**0.96± 0.1**
65%	Fast.ai	**0.99± 0.0**	0.64± 0.4	0.78± 0.3
	HAN	0.93± 0.0	0.67± 0.0	0.12± 0.1
	NIM	0.94± 0.0	0.67± 0.1	0.42± 0.1
	JELI	**0.99± 0.0**	**0.94± 0.0**	**0.94± 0.1**
80%	Fast.ai	**0.99± 0.0**	0.91± 0.1	0.77± 0.2
	HAN	0.96± 0.0	0.72± 0.0	0.20± 0.1
	NIM	0.93± 0.0	0.61± 0.1	0.19± 0.0
	JELI	**0.99± 0.0**	**0.94± 0.0**	**0.85± 0.2**

The NDCG at rank $n_i$ is averaged across users. NIM is NIMCGCN

Bold type is used for the highest value(s) in each experiment

#### Ablation study: both the structure and the joint learning are crucial to the performance

We perform the same type of experiments as in Sect. "JELI is robust in synthetic data sets across sparsity numbers" on several ablated versions of JELI to estimate the contribution of each part to the predictive performance. We introduce several JELI variants. First, we remove the structured and embedding part of the RHOFM classifier. FM is the regular second-order factorization machine of dimension *d* on 2*F*-dimensional input vectors, without structure on the coefficients (see Definition 1), whereas CrossFM2 is a more refined non-structured second-order factorization machine, where the feature pairwise interaction terms only comprise pairs of features on both the item and user vectors, that is, with notation from Definition 1

12 $$\begin{aligned} \text {CrossFM}_{(\omega ^0, \varvec{\omega }^1, \varvec{\omega }^2)}(\varvec{x}^i, \varvec{x}^u) \triangleq \omega ^0 + (\varvec{\omega }^1)^\intercal \begin{bmatrix}\varvec{x}^i\\ \varvec{x}^u\end{bmatrix} + \sum _{f \le F, f'> F} \langle \varvec{\omega }^2_{f}, \varvec{\omega }^2_{f'}\rangle \varvec{x}^i_{f} \varvec{x}^u_{f'-F}\;. \end{aligned}$$

Next, we also study methods featuring separate learning of the embeddings and the RHOFM classifier, named Separate Embedding Learning and Training algorithms (SELT). We consider different feature embedding types. SELT-PCAf uses the *d* principal component analysis (PCA) run on the concatenation of the item and user matrices along the column dimension, resulting in a $F \times (n_i+n_u)$ matrix. SELT-PCAf then infers feature embeddings based on each feature’s *d* first principal components. Another PCA-based baseline, SELT-PCAiu, applies the learned PCA transformation directly on item and user feature vectors to obtain item and user embeddings. Finally, the SELT-KGE approach completes the knowledge graph task to obtain item and user embeddings—without enforcing the feature-dependent structure—on the knowledge graph described in Definition 3 with an empty partial graph. Then, SELT-KGE uses those item and user embeddings to train the RHOFM classifier.

**Fig. 4:**
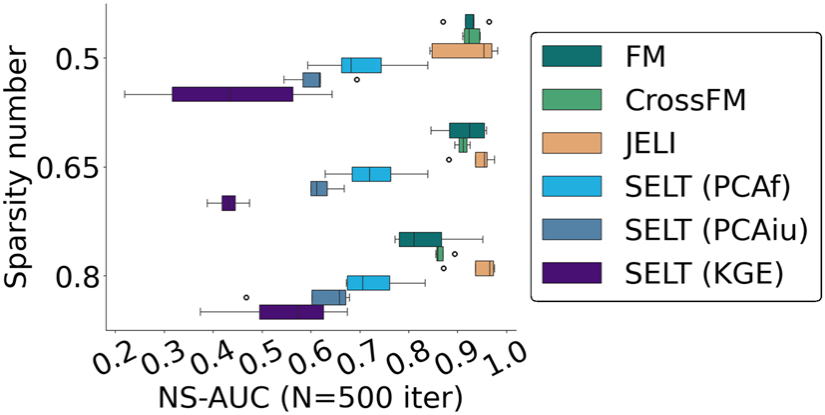
NS-AUC values across “first-order” synthetic data sets for sparsity numbers and 500 iterations for JELI and ablated variants. This shows that the most crucial part for a good predictive performance across sparsity numbers is the factorization machine

**Table 4:** Average metrics with standard deviations across 100 iterations and 5 “first-order” sets. The NDCG at rank $n_i$ is averaged across users. S indicates an instance of SELT

		AUC	NS-AUC	NDCG
50%	FM2	**0.99± 0.0**	0.92± 0.0	0.97± 0.0
	CrossFM2	**0.99± 0.0**	**0.93± 0.0**	**1.00± 0.0**
	S-PCAf	0.95± 0.0	0.70± 0.1	0.58± 0.2
	S-PCAiu	0.95± 0.0	0.61± 0.2	0.45± 0.2
	S-KGE	0.91± 0.0	0.43± 0.2	0.25± 0.2
	JELI	**0.99± 0.0**	0.92± 0.1	0.96± 0.0
65%	FM2	0.98± 0.0	0.91± 0.0	0.87± 0.1
	CrossFM2	**0.99± 0.0**	0.91± 0.0	**0.95± 0.0**
	S-PCAf	0.95± 0.0	0.73± 0.1	0.54± 0.2
	S-PCAiu	0.94± 0.0	0.62± 0.0	0.34± 0.1
	S-KGE	0.90± 0.0	0.43± 0.0	0.06± 0.0
	JELI	**0.99± 0.0**	**0.94± 0.0**	0.94± 0.1
80%	FM2	0.97± 0.0	0.84± 0.1	0.56± 0.1
	CrossFM2	0.98± 0.0	0.87± 0.0	0.74± 0.0
	S-PCAf	0.95± 0.0	0.73± 0.1	0.38± 0.1
	S-PCAiu	0.93± 0.0	0.62± 0.1	0.20± 0.0
	S-KGE	0.91± 0.0	0.55± 0.1	0.12± 0.1
	JELI	**0.99± 0.0**	**0.94± 0.0**	**0.85± 0.2**

Bold type is used for the highest value(s) in each experiment

The final results in Fig.4 and Table 4 show that the most crucial part for predictive performance across sparsity numbers is the factorization machine, which is unsurprising given the literature on factorization machines applied to sparse data. One can observe that separate embedding learning and factorization machine training leads to mediocre performance. The combination of a structured factorization machine and jointly learned embeddings, that is, JELI, gives the best performance and is even more significant as the set of known associations gets smaller (and the sparsity number is larger).

### Application to drug repurposing

We aim to predict new therapeutic indications, that is, novel associations between chemical compounds and diseases. The interpretability of the model for predicting associations between molecules and pathologies is crucial to encourage its use for health. In that case, higher-order factorization machines are very interesting models due to their inherent interpretability. However, particularly for the most recent drug repurposing datasets (*e*.*g*., TRANSCRIPT [43] and PREDICT [49]), the number of features ($F\approx 12,000$ and $F\approx 6,000$, respectively) is too large to effectively train a factorization machine due to the curse of dimensionality. Resorting to knowledge graphs then enables the construction of low-dimensional vector representations of these associations. Then, these representations are fed as input to the classifier during training instead of the initial feature vectors.

#### JELI is on par with state-of-the-art approaches on drug repurposing data sets

**Fig. 5:**
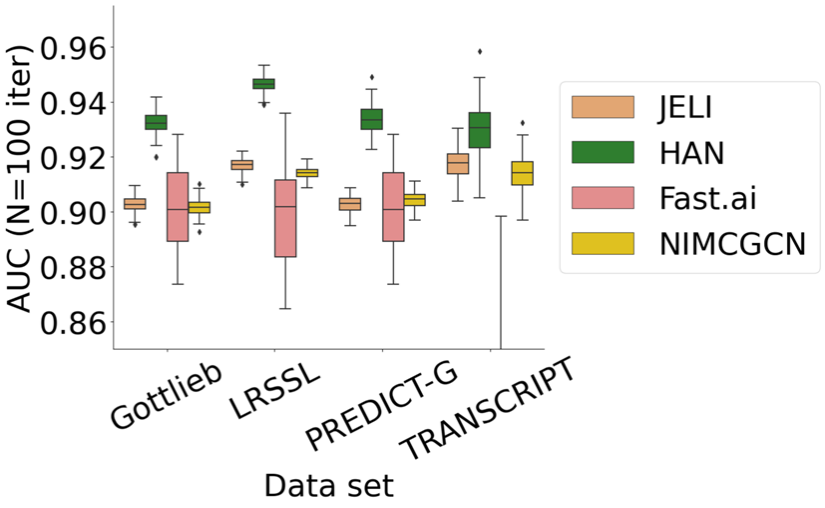
AUC values across drug repurposing data sets for 100 iterations for JELI and state-of-the-art embedding-based approaches

We now run JELI and the baseline algorithms tested in Sect. "JELI is robust in synthetic data sets across sparsity numbers" on Gottlieb [50] (named Fdataset in the paper), LRSSL [51], PREDICT-Gottlieb [52] and TRANSCRIPT [43] drug repurposing data sets which feature a variety of data types and sizes. Please refer to Appendix 4 for more information. Figure 5 and Table 5 report the validation metrics for each method’s 100 different training/testing splits with $d=15$. From those results, we can see that the performance of JELI is on par with the top algorithm, HAN, and sometimes outperforms it while providing interpretability.

For the sake of completeness, we also considered one of the most popular data sets for recommendation, called MovieLens [2], to better assess the performance of JELI for the general purpose of collaborative filtering. The goal is to predict if a movie should be recommended to a user, that is, if the user would rate this movie with more than 3 stars. The movie features are the year and the one-hot encodings of the movie genres, whereas the user features are the counts of each movie tag that this user has previously assigned. This experiment confirms that the performance of JELI is on par with the baselines, even in a non-biological setting. Please refer to Appendix 4 for more information.

**Table 5:** Average metrics with standard deviations across 100 iterations for each drug repurposing data set

		AUC	NS-AUC	NDCG
Gottlieb	Fast.ai	0.90± 0.0	0.50± 0.1	0.01± 0.0
	HAN	0.93± 0.0	0.67± 0.0	0.02± 0.0
	NIM	0.90± 0.0	0.51± 0.0	0.01± 0.0
	JELI	0.90± 0.0	0.52± 0.0	0.02± 0.0
LRSSL	Fast.ai	0.90± 0.0	0.49± 0.1	0.01± 0.0
	HAN	0.95± 0.0	0.69± 0.0	0.10± 0.0
	NIM	0.91± 0.0	0.53± 0.0	0.01± 0.0
	JELI	0.92± 0.0	0.51± 0.0	0.02± 0.0
PRED-G	Fast.ai	0.90± 0.0	0.50± 0.1	0.01± 0.0
	HAN	0.93± 0.0	0.68± 0.0	0.01± 0.0
	NIM	0.91± 0.0	0.49± 0.0	0.01± 0.0
	JELI	0.90± 0.0	0.47± 0.0	0.02± 0.0
TRANSC	Fast.ai	0.61± 0.1	0.57± 0.1	0.04± 0.0
	HAN	0.93± 0.0	0.61± 0.0	0.08± 0.0
	NIM	0.92± 0.0	0.57± 0.0	0.04± 0.0
	JELI	0.92± 0.0	0.56± 0.0	0.02± 0.0

The NDCG at rank $n_i$ is averaged across users. NIM is the algorithm NIMCGCN, TRANSC refers to the data set TRANSCRIPT, and PRED-G to the data set PREDICT-Gottlieb

#### JELI can integrate any graph prior on the TRANSCRIPT data set

**Fig. 6:**
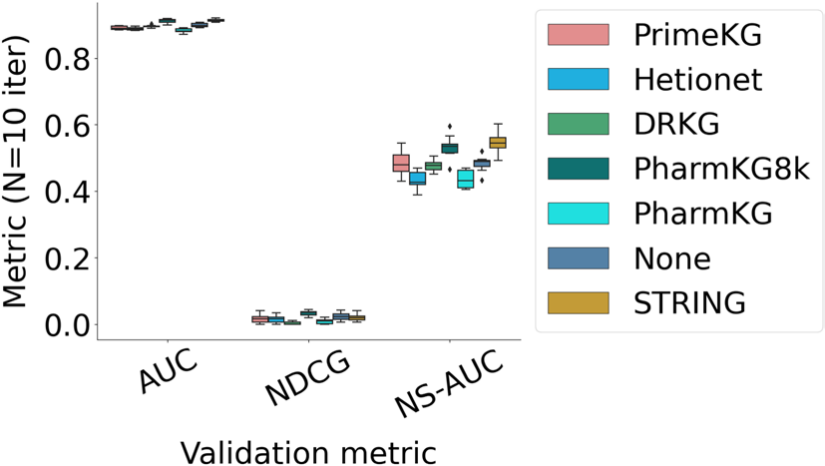
Predictive performance of JELI with different graph priors on different validation metrics

We now focus on the TRANSCRIPT data set, which involves gene activity measurements across $F=12,096$ genes for $n_i=204$ drugs and $n_u=116$ diseases. We compare the predictive power of JELI on the TRANSCRIPT data set with the default knowledge graph created by JELI (named “None” network, as we don’t rely on external sources of knowledge) and the default graph augmented with an external knowledge graph. The “None” network corresponds to the knowledge graph in Definition 3 with an empty partial graph. We considered as external knowledge graphs DRKG [53], Hetionet [54], PharmKG and PharmK8k (a subset of 8, 000 triplets) [41] and PrimeKG [42] as provided by the Python library PyKeen [55]. In addition, we also built a partial graph listing protein-protein interactions (where proteins are matched one-to-one to their corresponding coding genes) based on the STRING database [56]. The resulting accuracies in classification are shown on Figure 6 and Table 6. Most of the external graph priors significantly improve the classification accuracy, particularly the specific information about gene regulation (prior STRING). In Appendix 4, we also show that the graph priors’ performance correlates with a more frequent grouping of genes that belong to the same functional pathways. In Appendix 5, we perform a more thorough analysis of the specific case of melanoma and show that the predicted drug-disease associations and perturbed pathways allow us to recover some elements of the literature on melanoma.

**Table 6:** Average metrics with standard deviations across 10 iterations on the TRANSCRIPT data set for different graph priors

Graph prior	AUC	NS-AUC	NDCG
None	0.90± 0.01	0.48± 0.02	0.02± 0.01
DRKG	0.90± 0.00	0.48± 0.02	0.00± 0.00
Hetionet	0.89± 0.00	0.43± 0.02	0.01± 0.01
PharmKG	0.88± 0.01	0.43± 0.03	0.01± 0.01
PharmKG8k	0.91± 0.01	0.53± 0.03	0.03± 0.01
PrimeKG	0.89± 0.01	0.48± 0.03	0.02± 0.01
STRING	0.91± 0.00	0.55± 0.03	0.02± 0.01

## Discussion

This work proposes the JELI approach for integrating knowledge graph-based regularization into an interpretable recommender system. The structure incorporated into user and item embeddings considers numerical feature values in a generic fashion, which allows one to go beyond the categorical relations encoded in knowledge graphs without adding many parameters. This method allows us to derive item and user representations of fixed dimensions and score a user-item association, even on previously unseen items and users. We have shown the performance and the explainability power of JELI on synthetic and real-life data sets. The Python package that implements the JELI approach is available at the following open-source repository: github.com/RECeSS-EU-Project/JELI. Experimental results can be reproduced using code uploaded at github.com/RECeSS-EU-Project/JELI-experiments.

## Conclusions

This paper introduces and empirically validates our algorithmic contribution, JELI, for drug repurposing. JELI aims to provide straightforward interpretability in recommendations while integrating any graph information on items and users. However, there are a few limitations to the JELI approach. The first one is that JELI performs best on sparse user and item feature matrices, to exploit to the fullest the expressiveness of factorization machines. Moreover, this approach is quite slow compared to state-of-the-art algorithms since it simultaneously solves two tasks: the recommendation one on user-item pairs and the knowledge graph completion. We discuss the scalability of JELI with respect to various parameters in Appendix 6. However, this slowness is mitigated by the superior interpretability of JELI compared to the baselines. Furthermore, an interesting subsequent work would focus on integrating missing values into the recommendation problem. As it is, JELI ignores the missing features and potentially recovers qualitative item-feature –respectively, user-feature– links during the knowledge graph completion tasks. That is, provided an approach to quantify the strength of the link between an item and a feature, JELI might also be extended to perform an imputation of this item’s corresponding missing feature value.

## Figures and Tables

**Fig. 7:**
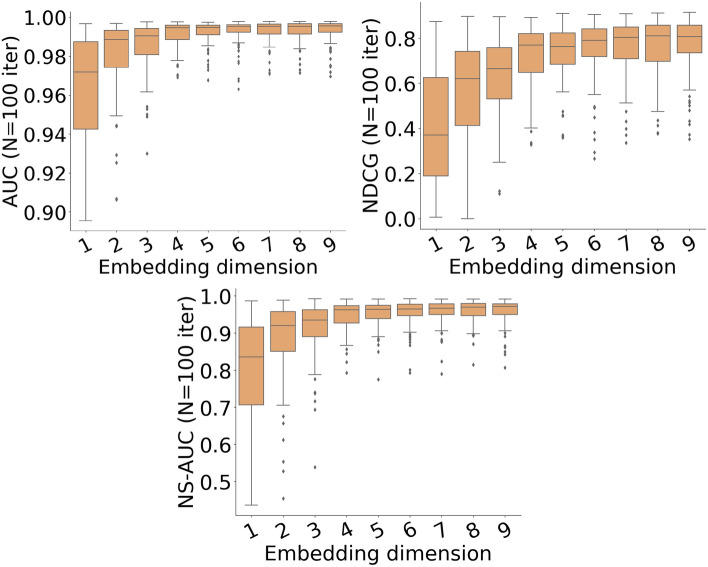
Validation metrics for JELI with different embedding dimension values *d* on a “first-order” synthetic data set with true dimension $d^\star =2$, across 100 iterations (random seeds for splitting between training and testing data sets)

**Fig. 8:**
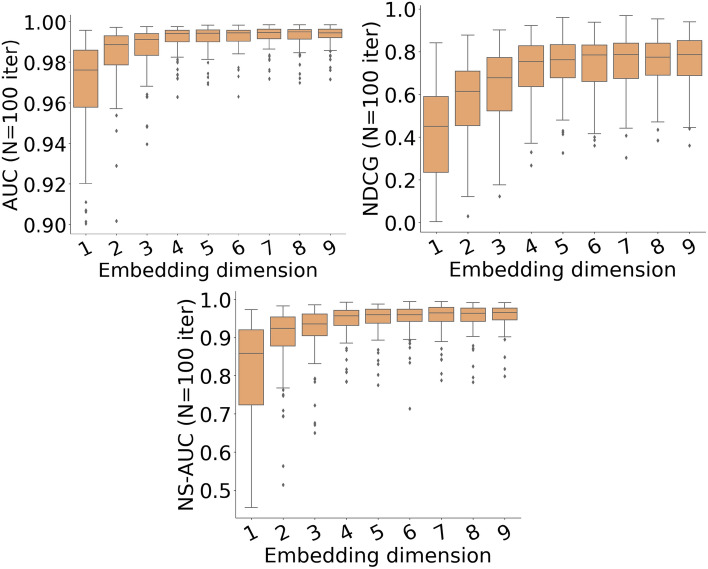
Validation metrics for JELI with different embedding dimension values *d* on a “first-order” synthetic data set with true dimension $d^\star =5$, across 100 iterations (random seeds for splitting between training and testing data sets)

**Fig. 9:**
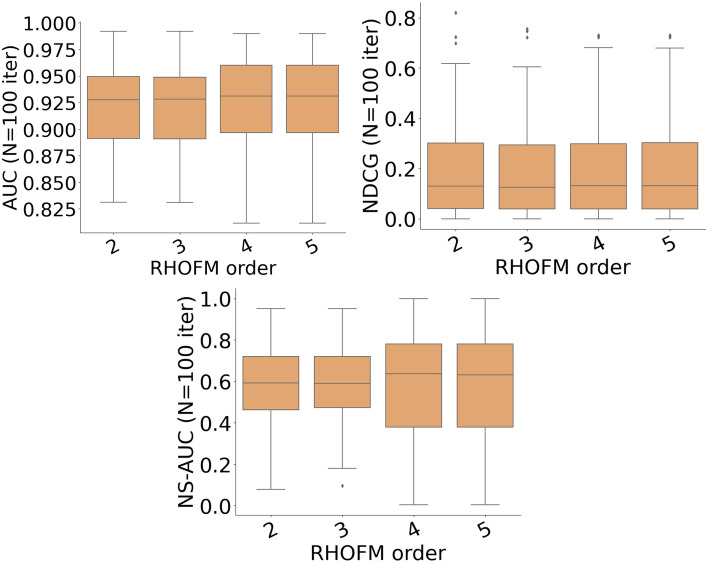
Validation metrics for JELI with different order *m* of the underlying RHOFM on a “second-order” synthetic data set across 100 iterations (random seeds for splitting between training and testing data sets)

**Fig. 10:**
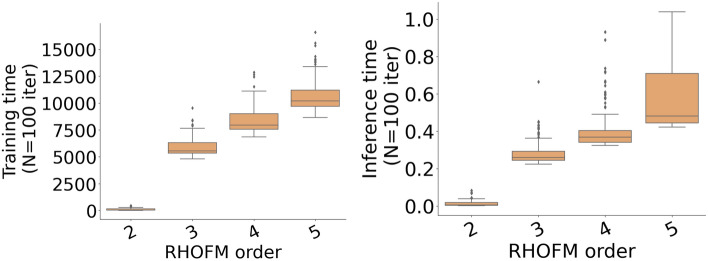
Training and inference (prediction across all pairs in the testing set) runtimes in seconds for JELI with different order *m* of the underlying RHOFM on a “second-order” synthetic data set across 100 iterations (random seeds for splitting between training and testing data sets)

**Fig. 11:**
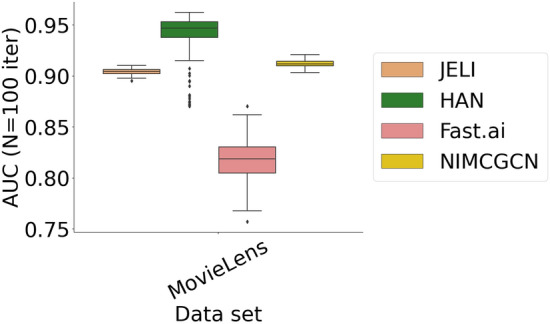
AUC values in the MovieLens data set and 100 iterations for JELI and state-of-the-art embedding-based approaches

**Fig. 12:**
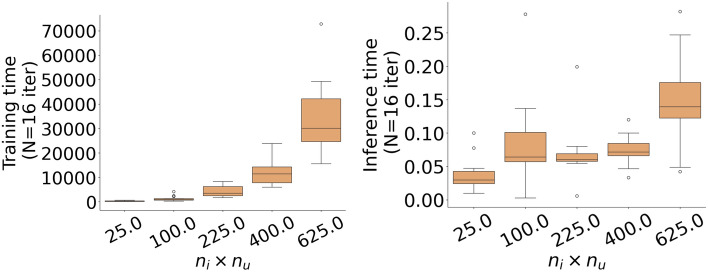
Training and inference (prediction across all pairs in the testing set) runtimes in seconds for JELI with different numbers of items and users $n_i=n_u \in \{5, 10, 15, 20, 25\}$, on a “second-order” synthetic data set across several iterations (random seeds for splitting between training and testing data sets)

**Fig. 13:**
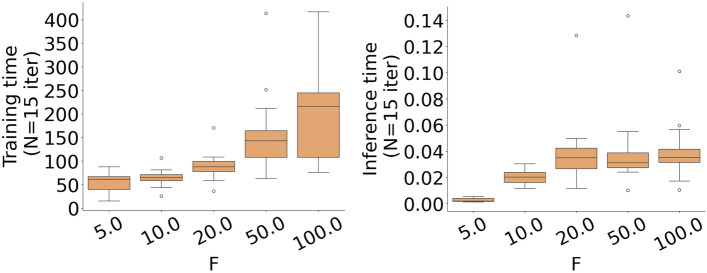
Training and inference (prediction across all pairs in the testing set) runtimes in seconds for JELI with different numbers of features $F \in \{5, 10, 20, 50, 100\}$, on a “second-order” synthetic data set across several iterations (random seeds for splitting between training and testing data sets)

**Fig. 14:**
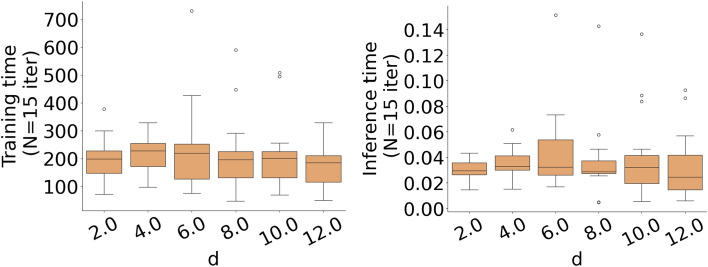
Training and inference (prediction across all pairs in the testing set) runtimes in seconds for JELI with different embedding dimensions $d \in \{2, 4, 6, 8, 10, 12\}$, on a “second-order” synthetic data set across several iterations (random seeds for splitting between training and testing data sets)

**Fig. 15:**
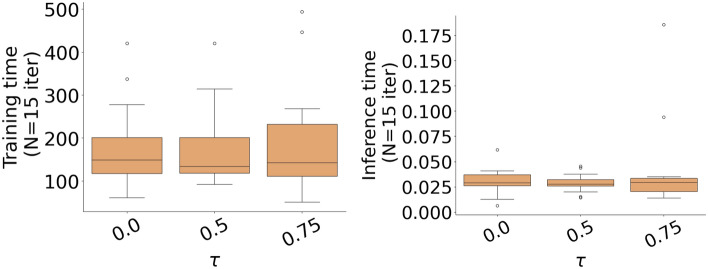
Training and inference (prediction across all pairs in the testing set) runtimes in seconds for JELI with different similarity thresholds $\tau \in \{0, 0.50, 0.7\}$, on a “second-order” synthetic data set across several iterations (random seeds for splitting between training and testing data sets)

**Fig. 16:**
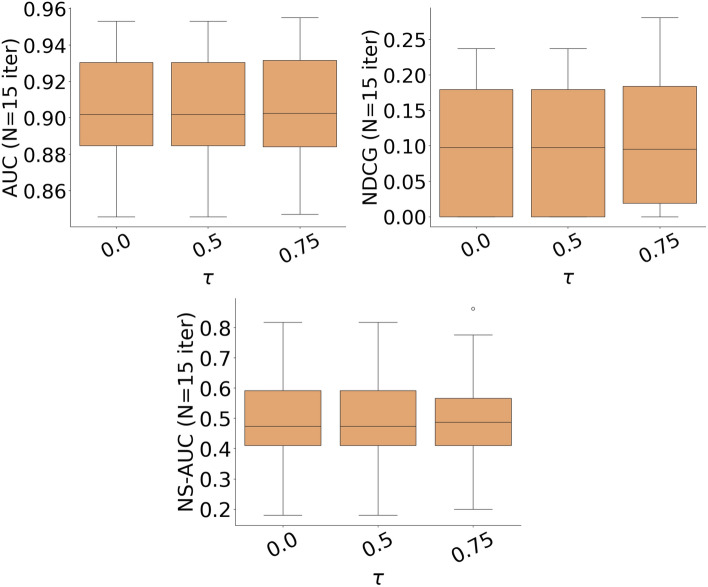
Validation metrics for JELI with different similarity thresholds $\tau$ on a “second-order” synthetic data set across several iterations (random seeds for splitting between training and testing data sets)

## Data Availability

The datasets analyzed during the current study are available through the stanscofi package and reported in Table C1 (github.com/RECeSS-EU-Project/stanscofi). The Python package that implements the JELI approach is available at the following open-source repository: github.com/RECeSS-EU-Project/JELI (DOI: 10.5281/zenodo.12193722). Experimental results can be reproduced using code uploaded at: github.com/RECeSS-EU-Project/JELI-experiments.

## Appendix 1: Explicit structure-dependent approximation of a RHOFM

Starting from the notation and the expression of an RHOFM introduced by Definition 2

A1 $$\begin{aligned} \text {RHOFM}_{\varvec{\theta }}(\varvec{x}^i, \varvec{x}^u)\triangleq & \omega ^0 + (\varvec{\omega }^1)^\intercal (\varvec{x}'^{iu})^\intercal \begin{bmatrix}\widetilde{W}^{iu}_\lambda \\ \widetilde{W}^{iu}_\lambda \end{bmatrix} \\ & + \sum _{2 \le t \le m} \varvec{\omega }^{2:m}_{t-1} \sum _{\begin{array}{c} f_1< \dots < f_t\\ f_1,\dots ,f_t \le 2F \end{array}} \left\langle \begin{bmatrix}\widetilde{W}^{iu}_\lambda \\ \widetilde{W}^{iu}_\lambda \end{bmatrix}_{f_1,:},\ldots , \begin{bmatrix}\widetilde{W}^{iu}_\lambda \\ \widetilde{W}^{iu}_\lambda \end{bmatrix}_{f_t,:} \right\rangle \varvec{x}'^{iu}_{f_1}\varvec{x}'^{iu}_{f_2} ... \varvec{x}'^{iu}_{f_t}\;. \nonumber \end{aligned}$$

Given the definition of $\widetilde{W}^{iu}_\lambda$, it is easy to see that

A2 $$\begin{aligned} (\varvec{x}'^{iu})^\intercal \begin{bmatrix}\widetilde{W}^{iu}_\lambda \\ \widetilde{W}^{iu}_\lambda \end{bmatrix} = (\varvec{x}^i)^\intercal \widetilde{W}^{iu}_\lambda + (\varvec{x}^u)^\intercal \widetilde{W}^{iu}_\lambda \approx s_W(\varvec{x}^i) + s_W(\varvec{x}^u) \;. \end{aligned}$$

Let us consider now the *t*-interaction term, for $t \ge 2$. For any set of *t* features $f_1, f_2, \dots , f_t$, using the notation $\varvec{x}^f \triangleq (\delta _{f_j=f})_{j \le |F|}$ and $f \% F$ as the remainder of the Euclidean division of *f* by *F*

A3 $$\begin{aligned} \left\langle \begin{bmatrix}\widetilde{W}^{iu}_\lambda \\ \widetilde{W}^{iu}_\lambda \end{bmatrix}_{f_1,:},\ldots , \begin{bmatrix}\widetilde{W}^{iu}_\lambda \\ \widetilde{W}^{iu}_\lambda \end{bmatrix}_{f_t,:} \right\rangle \varvec{x}'^{iu}_{f_1}\varvec{x}'^{iu}_{f_2}... \varvec{x}'^{iu}_{f_t}= & \sum _{k \le d} \left( \Pi _{l \le t} \ (\widetilde{W}^{iu}_\lambda )_{f_l \% F,k} \right) \left( \Pi _{j \le t} \ \varvec{x}'^{iu}_{f_j}\right) \qquad \\= & \sum _{k \le d} \left( \Pi _{l \le t} \ \varvec{x}^{f_l \% F} (\widetilde{W}^{iu}_\lambda )_{:,k} \right) \left( \Pi _{j \le t} \ \varvec{x}'^{iu}_{f_j}\right) \nonumber \\\approx & \sum _{k \le d} \left( \Pi _{l \le t} \ s_W(\varvec{x}^{f_l \% F})_k \right) \left( \Pi _{j \le t} \ \varvec{x}'^{iu}_{f_j}\right) \nonumber \\= & \sum _{k \le d} \Pi _{j \le t} \ \varvec{x}'^{iu}_{f_j} s_W(\varvec{x}^{f_j \% F})_{k} \nonumber \\= & \left\langle \varvec{x}'^{iu}_{f_1} s_W(\varvec{x}^{f_1 \% F}),\ldots , \varvec{x}'^{iu}_{f_t} s_W(\varvec{x}^{f_t \% F}) \right\rangle \;. \nonumber \end{aligned}$$

This leads to Eq. (5) in the main text.

## Appendix 2: Implementation of the joint training procedure in JELI

The training procedure iteratively updates across epochs and batches of triplets the feature embeddings $W \in \mathbb {R}^{F \times d}$, the MuRE-specific hyperparameters $R_r \in \mathbb {R}^{d \times d}$ for each relation *r* (9 in total by Definition 3), the biases $b \in \mathbb {R}^{|\mathcal {V}|}$, and the hyperparameters of the RHOFM $(\omega ^0, \varvec{\omega }^1, \varvec{\omega }^{2:m}) \in \mathbb {R} \times \mathbb {R}^d \times \mathbb {R}^{m-1}$, for a total of $(9d^2+|\mathcal {V}|)+(1+m+2Fd)=d(9d+2F)+|\mathcal {V}|+m+1$ parameter values. In practice, we implement this procedure using the PyKeen Python package [55], an Adam optimizer, and the PseudoTypedNegativeSampler class in PyKeen for the negative sampling to switch the head of triplets and then compute the soft margin ranking loss $\text {L}^\text {margin}$. The MuRE interaction class in PyKeen is modified to allow the computation of structured embeddings for items and users in the score.

Before training, we normalize the item and user feature matrices to cope with heterogeneous feature values. We replace missing values with zeroes, quantile normalize each feature and then normalize to $[-1,1]$ (with the function normalize$(\cdot , \text {norm}=\ell _1)$ from the Python package scikit-learn [57]).

We also force sparsity in feature values by adding a supplementary preprocessing layer which removes all “weak-signal” normalized values *v* such that $|v|<t \in (0,1)$ (thresholding with value $t=0.001$) or such that

B4 $$\begin{aligned} \inf _{v'} \left\{ \text {freq}(v') \le \frac{q}{2} \right\}< v < \inf _{v'} \left\{ \text {freq}(v') \ge \frac{1-q}{2} \right\} \ , \ q \in (0,1) \;. \end{aligned}$$

We use the latter method throughout the experimental study, with $q=0.9$. Note that those two approaches are equivalent for normally distributed frequencies of values.

## Appendix 3: Experimental details

**Table 7:** Overview of the drug repurposing data sets in the experimental study in Sect. "Results", with the number of items (drugs), item features, users (diseases), user features, positive and negative associations along with the corresponding sparsity number

Data set	$n_i$ $|\mathcal {F}_i|$	$n_u$ $|\mathcal {F}_u|$	Nb. positive	Nb. negative	sparsity (%)
Gottlieb	593 593	313 313	1,933	0	99.0
PREDICT-G	593 1,779	313 313	1,933	0	99.0
LRSSL	763 2,049	681 681	3,051	0	99.4
TRANSCRIPT	204 12,096	116 12,096	401	11	98.3

### Data preprocessing

All the data sets, including the synthetic ones, do not have any missing values. Before being fed to any classifier, the drug, and disease features are standard-normalized using class StandardScaler in scikit-learn [57].

### Drug repurposing data sets

The drug repurposing data sets were retrieved using the stanscofi Python package [3]. Table 7 shows their size and an overview of their contents. When items and users did not use the same set of features $\mathcal {F}_i$ and $\mathcal {F}_u$, we considered the disjoint union of the item and user feature sets $\mathcal {F}_i \cup \mathcal {F}_u$ by padding with zeroes whenever a feature was missing.

### Composition of the training sets in classification

The training and testing sets are split from the corresponding data set using function random_simple_split from package stanscofi [3] which splits the data into 80% and 20% blocks and prevents data leakage by carefully keeping separate folds (sets of triplets (drug, disease, annotation)) to construct the sets. Please refer to the related reference for more details.

### Synthetic dataset

In both considered types of synthetic data sets, as described in the main text at Sect. "Results", we first draw at random the item and user feature matrices $\widetilde{S}$ and $\widetilde{P}$ and feature embeddings $W^\star$ from a standard Gaussian distribution and set a fixed generating model $g_0$ for computing ground truth association scores based on item and user feature vectors. We now lay out how the sparsity in feature values (Sect. "Adapted Gaussian self-masking procedure") and in associations (Sect. "Enforcing the sparsity in associations") is implemented.

#### Adapted Gaussian self-masking procedure

As mentioned in the main text, we implemented a slightly modified version of the Gaussian self-masking procedure introduced in [47, Assumption 4] to generate not missing at random values. For each entity (item or user) $j \le n$, where $n \in \{n_i, n_u\}$ the number of entities, we denote $M_{j,f}$ the binary value which indicates whether the feature value $\varvec{x}^j_f$ is missing. For fixed feature-specific coefficients $K_f \in (0,1)$ for any feature $f \le F$, we then recall that the Gaussian self-masking mechanism is defined as

C5 $$\begin{aligned} \mathbb {P}(M_{j,1},\ldots , M_{j,F} \mid (\varvec{x}^j)_{j \le n}) = \Pi ^F_{f=1} K_f \exp \left( -\frac{1}{2}\frac{(\varvec{x}^j_f - \widetilde{\mu }_f)^2}{\widetilde{\sigma }_f^2} \right) \;,\end{aligned}$$

where $\widetilde{\mu }_f \triangleq \frac{1}{n}\sum _{l \le n} \varvec{x}^l_f \text { and } \widetilde{\sigma }_f^2 \triangleq \frac{1}{n-1}\sum _{l \le n} (\varvec{x}^l_f-\widetilde{\mu }_f)^2$. We want to ensure that the sparsity (*i*.*e*., the percentage of feature values set to zero) is at most at $10\%$. We then modify the Gaussian self-masking procedure as follows: after drawing at random the coefficients $(K_f)_{f \le F}$ and min–max normalizing them, we define the probability of the feature value associated with feature *f* of being missing as

C6 $$\begin{aligned} \mathbb {P}(M_{j,f} \mid (\varvec{x}^j_f)_{j \le n}) = 0.2 K_f \exp \left( -\frac{1}{2}\frac{(\varvec{x}^j_f - \widetilde{\mu }_f)^2}{\widetilde{\sigma }_f^2} \right) \;. \end{aligned}$$

We define then the final item and user feature matrices as$S \triangleq M^i \otimes \widetilde{S} \text { and } P \triangleq M^u \otimes \widetilde{P}\;,$ where $M^i$ and $M^u$ are drawn from a Gaussian self-masking procedure with respective input matrices *S* and *P*, and $\otimes$ is the element-wise matrix multiplication.

#### Enforcing the sparsity in associations

Given the final item and user feature matrices as defined in the last paragraph, we set the association score matrix $\widetilde{A}$ such that for each item *i* and user *u*, $\widetilde{A}_{i,u} \triangleq g_0(S_{:,i},P_{:,u})$. Then, we would like to ensure that the sparsity number –that is, the percentage of unknown user-item associations– is equal to $s \in (0.5, 1)$. If *t*(*s*) and $t'(s)$ are respectively the $\frac{100(1+s)}{2}^\text {th}$ and $\frac{100(1-s)}{2}^\text {th}$ quantiles of values in $\widetilde{A}$, then we define the final association matrix $A \in \{-1,0,+1\}^{n_i \times n_u}$ as

C7 $$\begin{aligned} \forall i \le n_i, \ \forall u \le n_u, \ A_{i,u} = {\left\{ \begin{array}{ll} \ +1 & \text { if } \widetilde{A}_{i,u} \ge t(s)\\ \ -1 & \text { if } \widetilde{A}_{i,u} \le t'(s) \\ \ 0 & \text { otherwise} \end{array}\right. }\;. \end{aligned}$$

### Training in separate embedding/RHOFM learning approaches (SELT or factorization machines)

To train the corresponding baseline models, we reimplement a training procedure with Python package PyTorch [58] equivalent to the PyKeen fit function (that is, using the margin ranking loss, the same parameters to the Adam optimizer, and a negative sampler which associates 3 negative samples to each positive sample in a batch. The negative sampler uses negative_sampling from the Python package PyTorch-Geometric [59].

In SELT-KGE—learning embeddings based on a knowledge graph completion task and then feeding them to the RHOFM solo training procedure—we use the same parameters as in JELI to a PyKeen training procedure to learn the embeddings.

## Appendix 4: Supplementary experiments

All experiments, including those shown in the main text, were run on remote cluster servers from Inria Saclay (processor QEMU Virtual v2.5+, 48 cores @2.20GHz, RAM 500GB).

### Parameter impact of the embedding dimension *d*

We performed two different tests on synthetic data sets. Considering the “first-order” type of synthetic data sets, we applied JELI with different embedding dimension parameters $d \in \{1,2,3,4,5,6,7,8,9\}$ and measured the corresponding AUC, NDCG and NS-AUC values on a synthetic data set with true dimension $d^\star =2$ (Fig. 7) and $d^\star =5$ (Fig. 8). It can be noticed that JELI remains robust to the value of *d* if we select a value of *d* larger than the true value $d^\star$.

### Parameter impact of the order *m* of the RHOFM

With varying *m* in $\{2,3,4,5\}$, we report the classification performance across 100 randomly generated synthetic data sets ($F=10$, $n_u=n_i=32$, $d=2$). The synthetic data sets correspond to the “second-order” data generation procedure, where considering higher-order interactions makes the most sense. Figure 9 shows the classification performance (AUC, NDCG, and NS-AUC) across orders, whereas Fig. 10 displays the training and inference runtime (in seconds) across orders.

As expected, the classification performance is noticeably better as the order increases (see Fig. 9). However, JELI pays a steep price for the training cost. The expression of a RHOFM for $m=2$ can be transformed into a quickly evaluated vectorized expression. However, we use the dynamic programming algorithms from [30] for $m>2$, where the time complexity is visibly linear in the order *m* (see Fig. 10). Linearity in *m* is already a good result. However, it accounts for a significant difference in computational cost compared to the case $m=2$, even for a few features and samples.

### Classification performance in the MovieLens data set [2]

The recommendation problem in MovieLens [2] is to predict whether a movie should be recommended to a user, that is, if the user would rate this movie with more than 3 stars. The movie features are the year and the one-hot encodings of the movie genres, whereas the user features are the counts of each movie tag that this user has previously assigned. We iterate this experiment for each algorithm (JELI and baselines) 100 times, and report the corresponding results in Fig. 11 and Table 8.

This experiment confirms that the performance of JELI is on par with the baselines, even in a non-biological setting.

**Table 8:** Average metrics with standard deviations across 100 iterations for the MovieLens data set [2]

		AUC	NS-AUC	NDCG
MovieLens	Fast.ai	0.82± 0.0	0.52± 0.0	0.15± 0.0
	HAN	0.94± 0.0	0.51± 0.1	0.11± 0.1
	NIM	0.91± 0.0	0.54± 0.0	0.12± 0.0
	JELI	0.90± 0.0	0.42± 0.0	0.09± 0.0

The NDCG at rank $n_i$ is averaged across users. NIM is the algorithm NIMCGCN

### Comparing gene embeddings and functional pathways

We want to measure how meaningful the gene embeddings are compared to existing annotated functional pathways. The way we approached this was to show that the gene embeddings successfully grouped genes that are known to belong to the same functional group. We focused on the well-known 50 Hallmark functional families [60] (also called gene sets), which documents 50 biological pathways regrouping several human genes. The main issue is that a gene can appear in several families simultaneously, preventing classical clustering validation measures such as Adjusted Rand Index (ARI) [61]. 49 of these groups feature at least one gene in the TRANSCRIPT data set, and 3497 genes out of the $F=12,096$ genes are present in at least one gene set in Hallmark.

First, we checked that the gene embeddings obtained for each graph prior had low variance, that is, the average variance of the value for a gene ($F=12,096$ in the TRANSCRIPT data set) and dimension ($d=50$) across iterations of JELI is low. See Table 9. Then, we considered the “average” embedding for each gene, obtained by averaging values per gene and per embedding dimension across iterations of JELI.

**Table 9:** Average minimum, mean, maximum, and variance, rounded to the third decimal place, of the embedding value per feature/gene ($F=12,096$) and per embedding dimension ($d=50$) across 10 iterations of JELI

	None	DRKG	Hetionet	PharmKG8k	PharmKG	PrimeKG	STRING
Minimum	−0.022	−0.462	−0.455	−0.457	−0.453	0.022	−0.531
Mean	−0.004	0.001	0.006	0.125	−0.003	0.004	−0.017
Maximum	0.014	0.464	0.462	0.453	0.457	0.022	0.512
Variance	0.0	0.129	0.126	0.125	0.218	0.0	0.109

Second, we defined a measure similar to ARI, which considers that the functional gene sets are not distinct. Given the gene sets $\mathcal {G}=\{G_1, G_2, \dots , G_{49}\}$ and the clustering $\mathcal {C}=\{C^p_1, C^p_2, \dots , C^p_K\}$ obtained by running a clustering algorithm on the gene embeddings for each graph prior *p*, the two sources of agreement between $\mathcal {G}$ and $\mathcal {C}$ are (a) when two genes that belong to a same functional group $G_l$ are clustered together in cluster $C^p_k$; (b) when two genes which never belong to the same functional set are not clustered together. Then, we denote $a_{i,j} \in \{0,1\}$ the variable which only takes the value 1 if and only if gene *i* and gene *j* match case (a); we similarly define $b_{i,j} \in \{0,1\}$. Then, we define a fuzzy Rand index (that allows overlaps) as

D8 $$\begin{aligned} \text {RI}(\mathcal {G}, \mathcal {C}) \triangleq 2\frac{\sum _{i,j \le F} a_{i,j}+b_{i,j}}{F(F-1)}\;. \end{aligned}$$

We add the correction for chance to obtain the “fuzzy” ARI. We define $g_{l,k} = \sum _{g \le F} \delta (g \in \mathcal {G}_l \cap \mathcal {C}_k)$, where $\delta (c) \in \{0,1\}$ is positive if and only if condition *c* is satisfied. Then, using $a_l \triangleq \sum _{k \le K} g_{l,k}$ and $b_k \triangleq \sum _{l \le 49} g_{l,k}$

D9 $$\begin{aligned} \text {ARI}(\mathcal {G}, \mathcal {C}) \triangleq \frac{N - 2\frac{N_a \times N_b}{F(F-1)}}{\frac{1}{2}(N_a+N_b) - 2\frac{N_a \times N_b}{F(F-1)}}\;, \end{aligned}$$

where $$N_a \triangleq \frac{1}{2}\sum _{l \le 49} a_l(a_l-1)$$, $$N_b \triangleq \frac{1}{2}\sum _{k \le K} b_k(b_k-1)$$ and $$N \triangleq \frac{1}{2}\sum _{l \le 49, k \le K} g_{l,k}(g_{l,k}-1)$$.

**Table 10:** Fuzzy ARI value, rounded to the third decimal place, computed between the 49 functional gene sets from Hallmark [60] and the clustering of gene embeddings for each graph prior and type of clustering

ARI	None	DRKG	Hetionet	PharmKG8k	PharmKG	PrimeKG	STRING
K-means	−0.048	−0.049	−0.051	−0.047	−0.049	−0.048	−0.050
HDBSCAN	−0.015	0.000	−0.087	0.032	0.015	0.013	0.041

We tested two clustering algorithms: the (greedy) K-means++, for which we provide the number of functional groups ($K=49$) as the number of expected clusters. This allows us to compare the “ARI” values across graph priors. However, this choice prevents us from forming natural embedding clusters, as the constraint on the number of clusters might lead to low clustering quality. Then we consider as second choice the density-based clustering algorithm HDBSCAN [62], which is adapted to cases where the number of clusters is not provided, for which we set the minimal distance between two points as the 25$^\text {th}$ percentile of pairwise distances between gene embeddings. The resulting ARI values are displayed in Table 10.

Then, we can compute Spearman’s $\rho$ correlation values with the average accuracy values for each graph prior in Table 6. For the K-means clusterings, we got $\rho _\text {AUC,ARI} = 0.18$, $\rho _\text {NDCG,ARI} = 0.29$ and $\rho _\text {NS-AUC,ARI} = 0.32$, whereas, for the HDBSCAN clusterings, we obtained $\rho _\text {AUC,ARI} = 0.50$, $\rho _\text {NDCG,ARI} = 0.50$ and $\rho _\text {NS-AUC,ARI} = 0.64$. Most likely, there is a positive correlation between grouping genes by their functional pathways and classification accuracy for the drug repurposing task on the TRANSCRIPT data set. The smaller correlation values for K-means clusterings might stem from the ARI values being typically lower because we forced the creation of $K=49$ clusters instead of going for more naturally shaped clusters.

## Appendix 5: Drug repurposing and pathway enrichment for melanoma

We focus on melanoma (MedGen Concept ID C0025202), a type of skin cancer stemming from the overproliferation of melanocytes. Melanoma is a disease for which prior literature is abundant and for which resistance to standard-of-care chemotherapies (cell checkpoint inhibitors) is documented. This makes melanoma a good target for drug repurposing, as an illustration.

Without access to a wet lab, we proceed with statistical analyses and a literature review. To do so, we run JELI on the TRANSCRIPT data set (that contains melanoma) with the STRING prior, order $m=2$, and coefficients $\omega _0=1$, $\omega _1 ={\textbf {1}}_d$, and $\omega ^{2:m}=1$.

### Exploration of novel drug recommendations for melanoma

First, we explore novel treatment recommendations for melanoma. In Table 11, we report all the drug recommendations with predicted recommendation scores higher than 0.920 for melanoma and the recommendation score for the only drug annotated positive for melanoma in the TRANSCRIPT data set (Lidocaine). No drug is negatively annotated for melanoma in the data set. The minimum recommendation score across drugs and diseases is 0.217. Scores are rounded to the closest third decimal place. We performed a literature search on each of these non-annotated chemical compounds concerning melanoma (the full table of information about those drugs is added to the appendix of the manuscript).

**Table 11:** Drug recommendations in decreasing order of recommendation score (rounded to the closest third decimal place) for melanoma after running JELI on the TRANSCRIPT data set

DrugBank ID	Drug name	Score	Label	Drug family	ATC
DB00928	Azacitidine	0.927	0	Pyrimidine nucleoside analogue	L01BC07
DB00331	Metformin	0.926	0	Biguanide antihyperglycemic	A10BA02
DB01067	Glipizide	0.924	0	Sulfonylurea medication	A10BB07
DB00685	Trovafloxacin	0.924	0	Broad spectrum antibiotic	unknown
DB01197	Captopril	0.924	0	ACE inhibitor	C09AA01
DB00983	Formoterol	0.924	0	Long-acting beta2-adrenergic	R03CC15
				receptor agonist	
DB00526	Oxaliplatin	0.921	0	Platinum-based chemotherapy	L01XA03
				agent	
DB01064	Isoprenaline	0.920	0	Catecholamine non-selective	C01CA02
				beta-adrenergic agonist	
DB00281	Lidocaine	0.704	1	Tertiary amine class Ib	C05AD01
				antiarrhythmic agent	

The identifiers and drug families come from DrugBank [63], whereas the Anatomical Therapeutic Code (ATC) is extracted from PubChem [64]. The ATC classifies drugs into hierarchical classes depending on their mechanisms of action. The label (0: unknown, 1: positive) in the TRANSCRIPT data set corresponds to the initial drug-disease class for melanoma used for the training of JELI

A literature search run on the top 0-labeled drugs in the 2019-2024 period showed that the top two drugs could have a therapeutic impact on melanoma patients, alone or in combination, as illustrated by several published studies. For further details, see the supplementary *Melanoma.csv* uploaded on the GitHub repository github.com/RECeSS-EU-Project/JELI-experiments. Too few studies were published to make conclusive comments on the remainder of the drugs. However, some of those compounds have a mechanism of action related to the top two drugs (DNA damage or insulin release), as demonstrated by their Anatomical Therapeutic Chemical (ATC) code.

### Pathway enrichment analysis for melanoma

Second, we use the importance scores to run a pathway enrichment analysis to determine which functional pathways are the most discriminative for melanoma treatment recommendations. We retrieved the feature embeddings computed by JELI and the feature vector associated with melanoma in the TRANSCRIPT data set. The resulting disease-specific importance scores are the element-wise product (across features) and then sum (across embedding dimensions) of the feature embeddings learned by JELI and the feature vector associated with the disease, as in Result 1. We obtained a list of 12,096 feature/gene scores for melanoma, among which 593 are non-zero, comprised in the interval [−2.550, 3.014].

Then, we run a Gene Set Enrichment Analysis (GSEA) [36], one of the most commonly used pathway enrichment methods, on this list of scores. We use the following parameters: size of enrichment sets: [5; 2,000], 10,000 permutations, maximum false discovery rate on enrichment sets: 20%. We used the web application WebGestalt [65], accessed on October 30th, 2024. This analysis returns Table 12 against annotation sets from KEGG gene sets (as available on the WebGestalt app).

**Table 12:** Enrichments from the Gene Set Enrichment Analysis (GSEA) run on the melanoma-related importance scores

Enriched KEGG category	Enrichment score	Normalized enrichment	False discovery rate
		score	
Phenylalanine, tyrosine	−0.996	−7.661	1,6%
and tryptophan biosynthesis			

Values are rounded to the closest third decimal place

The “Phenylalanine, tyrosine and tryptophan biosynthesis” pathway seems closely associated with melanoma. Tyrosine, phenylalanine (a precursor for tyrosine), and tryptophan are aromatic $\alpha$-amino acids [66]. This pathway is negatively enriched in melanoma, as shown by the (normalized) enrichment score. That is, the pathway’s activity should be decreased in melanoma patients. Some prior works favor this hypothesis for the metabolism of tryptophan [67, 68]. Moreover, [69, 70] also reported the effect of low phenylalanine and tyrosine intake on melanoma patients.

For the TRANSCRIPT data set, note that only gene expression level activity is considered to predict drug-disease associations, ignoring all post-transcriptional and epigenomic mechanisms, which often also control the therapeutic efficacy of a drug. As such, the recommended drugs and enriched categories might only hold at the level of transcriptional activity.

## Appendix 6: Scalability of the JELI algorithm

The complexity of the JELI algorithm is mainly driven by the computational time needed to evaluate the RHOFM and the size of the knowledge graph. Remember that we denote $n_i$ the number of items, $n_u$ the number of users, *F* the number of disjoint item and user features, *d* the embedding dimension and *m* the order of the RHOFM.

***About the evaluation of the RHOFM on all possible associations.*** The dynamic programming algorithms in [30] for $m>2$ and the fast, vectorized, formula for $m=2$, the time complexity for the evaluation of the RHOFM is of $\mathcal {O}(n_in_uFdm)$. The number of parameter values in the RHOFM is $1+m+(F+1)d$ (see Definition 2).

***About the knowledge graph.*** In the absence of an initial partial graph, the number of nodes is $n_i + n_u + F$, and the number of edges is at most $n_i \times n_u + n_u(n_u-1)/2 + n_i(n_i-1)/2 + F(n_i+n_u)$ (see Definition 3). In practice, those costs are much smaller, as the data sets are often very sparse, meaning that the number of known positive or negative associations is lesser than $n_i \times n_u$, and the number of non-zero features is smaller than $F(n_i+n_u)$. Finally, the hyperparameter $\tau$ for the similarity threshold on edges allows us to restrict the number of user-user and item-item similarities, lesser than $n_u(n_u-1)/2$ and $n_i(n_i-1)/2$, respectively.

***About the empirical performance.*** We have performed scalability experiments, considering a “second-order” synthetic data set (as described in Sect. Results) with the following default values: $n_i=32$, $n_u=32$, $F=100$, and running JELI with default parameters $d=2$, $\tau =0.75$, $m=2$. We vary one data or algorithm-related parameter at a time. Below, we display the boxplots of training and inference times in seconds across several iterations (random seeds) for all parameter values.

First, from Fig. 12, we observe that the training time is roughly polynomial in $n_i \times n_u$, as we have to run one evaluation of the RHOFM for drug-disease associations and compute the MuRE score for drug-drug and disease-disease similarities. The inference/prediction time on drug-disease associations is also increasing in $n_i \times n_u$, but JELI only enumerates over drug-disease associations from the much smaller testing set.

As for the number of users/items, Fig. 13 shows that the training time is also polynomial in the number of features, as JELI computes the MuRE score for feature-feature, drug-feature and disease-feature potential edges in the knowledge graph. Note that if the feature matrices are sparse (meaning that they comprise numerous zeroes, which is not the case in these synthetic data sets), JELI might be faster even though the number of features is more significant. Similarly, the computational runtime at inference time is increasing as F increases, but less dramatically.

Regarding the embedding dimension, for small values of *d*, the training and inference times are roughly constant as shown by Fig. 14. The reason might be linked to embeddings only appearing in the efficient computations related to the RHOFM (that is, the drug-disease edges).

Moreover, in Fig. 15, the training and inference times are shown to be slightly greater as the threshold $\tau$ increases. This is consistent with the fact that the number of present edges in the knowledge graph decreases with $\tau$ increasing, which leads JELI to compute more MuRE scores related to those “missing” drug–drug and disease–disease edges. However, the meaning of those edges controlled by $\tau$ is that two drugs or two diseases are similar, so $\tau$ should not be too low to provide JELI with meaningful information.

Finally, in Fig. 10, we observed that the computational complexity of JELI is roughly linear in the order of the RHOFM $m>2$. For $m=2$, a vectorized, faster formula for the expression of the RHOFM is available.

## Appendix 7: Selection of hyperparameters

This section focuses on the choice of important hyperparameters in the JELI algorithm: the embedding dimension *d*, the similarity threshold $\tau$ and the order of the RHOFM *m*.

First, as shown in Figs. 7 and 8, the classification performance reaches a plateau when the embedding dimension *d* is large enough. In those synthetic experiments, the plateau starts at $d=d^\star$, where $d^\star$ is the dimension used to generate the synthetic observations. The classification performance might decrease linearly when *d* is smaller than that critical value. This applies to any classification metric we use (AUC, NS-AUC, NDCG). Then, in those synthetic experiments, $d^\star$ achieves the best performance-efficiency tradeoff on the synthetic data set. For data not generated by a RHOFM or any linear model, this critical value corresponds to a dimension $1< d < F$, which can capture all relevant information from the set of features. A grid search on a subset of data can find such a value.

Second, in Definition 3, as the similarity threshold $\tau$ decreases, the number of edges in the knowledge graph increases by an additive factor from 0 to $n_u(n_u-1)/2 + n_i(n_i-1)/2$, where $n_u$ is the number of users and $n_i$ the number of items. However, we expect that adding stronger similarity and higher-quality information (that is, a high value of $\tau$) will generally lead to better performance (which seems to be confirmed by Fig. 16). A reasonable threshold for significant positive similarity between two elements is $\tau =0.75$ in the cosine score between two drug or disease feature vectors. This value can be a good tradeoff between adding enough user and item similarity information and the size of the knowledge graph.

Finally, as shown by Fig. 9, a higher order *m* of the RHOFM generally leads to better classification performance, but the computational cost when $m>2$ might be prohibitive (see Fig. 10). $m=2$ is recommended in practice, as the gain in performance is lower than the computation cost for higher-order RHOFMs

## Footnotes

1 Otherwise, one can join the two feature matrices and replace missing feature values by zeroes.

2 Up to the approximation made in $s_W(\widetilde{\varvec{x}}^{iu}) \approx \widetilde{\varvec{x}}^{iu} \widetilde{W}^{iu}_\lambda$.

## References

[1] Bell RM, Koren Y, Volinsky C. All together now: a perspective on the Netflix prize. Chance. 2010;23(1):24–9.

[2] Harper FM, Konstan JA. The movielens datasets: history and context. ACM Trans Interact Intell Syst. 2015;5(4):1–19.

[3] Réda C, Vie JJ, Wolkenhauer O. stanscofi and benchscofi: a new standard for drug repurposing by collaborative filtering. J Open Sour Softw. 2024;9(93):5973.

[4] Rendle S, Freudenthaler C, Gantner Z, Schmidt-Thieme L. BPR: Bayesian personalized ranking from implicit feedback. arXiv preprint arXiv:1205.2618. 2012.

[5] Chin WS, Yuan BW, Yang MY, Zhuang Y, Juan YC, Lin CJ. LIBMF: a library for parallel matrix factorization in shared-memory systems. J Mach Learn Res. 2016;17(86):1–5.

[6] Sra S, Dhillon I. Generalized nonnegative matrix approximations with Bregman divergences. Advances in neural information processing systems. 2005;18.

[7] Golub G, Kahan W. Calculating the singular values and pseudo-inverse of a matrix. J Soc Ind Appl Mathe Ser B Numer Anal. 1965;2(2):205–24.

[8] Howard J, et al.: Fast.ai. GitHub. https://github.com/fastai/fastai.

[9] Batmaz Z, Yurekli A, Bilge A, Kaleli C. A review on deep learning for recommender systems: challenges and remedies. Artif Intell Rev. 2019;52:1–37.

[10] Li J, Zhang S, Liu T, Ning C, Zhang Z, Zhou W. Neural inductive matrix completion with graph convolutional networks for miRNA-disease association prediction. Bioinformatics. 2020;36(8):2538–46. 31904845 10.1093/bioinformatics/btz965

[11] Réda C, Vie JJ, Wolkenhauer O. Comprehensive evaluation of collaborative filtering in drug repurposing. HAL preprint HAL-04626970. 2024. 10.1038/s41598-025-85927-x PMC11751339 39837888

[12] Rendle S, Factorization machines. In: IEEE International conference on data mining. IEEE. 2010;2010:995–1000.

[13] Lundberg SM, Lee SI. A unified approach to interpreting model predictions. Advances in neural information processing systems. 2017;30.

[14] Ribeiro MT, Singh S, Guestrin C. “Why should I trust you?” Explaining the predictions of any classifier. In: Proceedings of the 22nd ACM SIGKDD international conference on knowledge discovery and data mining; 2016. p. 1135–1144.

[15] Swamy V, Radmehr B, Krco N, Marras M, Käser T. Evaluating the explainers: black-box explainable machine learning for student success prediction in MOOCs. arXiv preprint arXiv:2207.00551. 2022.

[16] Fokkema H, de Heide R, van Erven T. Attribution-based explanations that provide recourse cannot be robust. J Mach Learn Res. 2023;24(360):1–37.

[17] Nickel M, Tresp V, Kriegel HP, et al. A three-way model for collective learning on multi-relational data. ICML. 2011;11:3104482–584.

[18] Wang Q, Mao Z, Wang B, Guo L. Knowledge graph embedding: a survey of approaches and applications. IEEE Trans Knowl Data Eng. 2017;29(12):2724–43.

[19] Bordes A, Usunier N, Garcia-Duran A, Weston J, Yakhnenko O. Translating embeddings for modeling multi-relational data. Advances in neural information processing systems. 2013;26.

[20] Yang B, Yih Wt, He X, Gao J, Deng L. Embedding entities and relations for learning and inference in knowledge bases. arXiv preprint arXiv:1412.6575. 2014.

[21] Dettmers T, Minervini P, Stenetorp P, Riedel S. Convolutional 2d knowledge graph embeddings. In: Proceedings of the AAAI conference on artificial intelligence. vol. 32; 2018.

[22] Sun Z, Deng ZH, Nie JY, Tang J. Rotate: Knowledge graph embedding by relational rotation in complex space. arXiv preprint arXiv:1902.10197. 2019.

[23] Balazevic I, Allen C, Hospedales T. Multi-relational poincaré graph embeddings. Advances in Neural Information Processing Systems. 2019;32.

[24] Ali M, Berrendorf M, Hoyt CT, Vermue L, Galkin M, Sharifzadeh S, et al. Bringing light into the dark: a large-scale evaluation of knowledge graph embedding models under a unified framework. IEEE Trans Pattern Anal Mach Intell. 2021;44(12):8825–45. 10.1109/TPAMI.2021.3124805 34735335

[25] Wu J, Shi W, Cao X, Chen J, Lei W, Zhang F, et al. DisenKGAT: knowledge graph embedding with disentangled graph attention network. In: Proceedings of the 30th ACM international conference on information & knowledge management; 2021. p. 2140–2149.

[26] Cvetkov-Iliev A, Allauzen A, Varoquaux G. Relational data embeddings for feature enrichment with background information. Mach Learn. 2023;112(2):687–720.

[27] Wu Y, Wang Z. Knowledge graph embedding with numeric attributes of entities. In: Proceedings of the third workshop on representation learning for NLP; 2018. p. 132–136.

[28] Vie JJ, Kashima H. Knowledge tracing machines: factorization machines for knowledge tracing. In: Proceedings of the AAAI conference on artificial intelligence. vol. 33; 2019. p. 750–757.

[29] Guo H, Tang R, Ye Y, Li Z, He X. DeepFM: a factorization-machine based neural network for CTR prediction. arXiv preprint arXiv:1703.04247. 2017.

[30] Blondel M, Fujino A, Ueda N, Ishihata M. Higher-order factorization machines. Advances in Neural Information Processing Systems. 2016;29.

[31] Breiman L. Random forests. Mach Learn. 2001;45:5–32.

[32] Marton S, Lüdtke S, Bartelt C, Stuckenschmidt H. GradTree: learning axis-aligned decision trees with gradient descent. In: Proceedings of the AAAI conference on artificial intelligence. vol. 38; 2024. p. 14323–14331.

[33] Wachter S, Mittelstadt B, Russell C. Counterfactual explanations without opening the black box: automated decisions and the GDPR. Harv JL & Tech. 2017;31:841.

[34] Shih A, Choi A, Darwiche A. A symbolic approach to explaining Bayesian network classifiers. arXiv preprint arXiv:1805.03364. 2018.

[35] Darwiche A, Hirth A. On the reasons behind decisions. arXiv preprint arXiv:2002.09284. 2020.

[36] Subramanian A, Tamayo P, Mootha VK, Mukherjee S, Ebert BL, Gillette MA, et al. Gene set enrichment analysis: a knowledge-based approach for interpreting genome-wide expression profiles. Proc Natl Acad Sci. 2005;102(43):15545–50. 16199517 10.1073/pnas.0506580102 PMC1239896

[37] Li J, Zhang C, Zhou JT, Fu H, Xia S, Hu Q. Deep-LIFT: deep label-specific feature learning for image annotation. IEEE Trans Cybern. 2021;52(8):7732–41. 10.1109/TCYB.2021.3049630 33566780

[38] Amoukou SI, Brunel NJ. Consistent Sufficient Explanations and Minimal Local Rules for explaining regression and classification models. arXiv preprint arXiv:2111.04658. 2021.

[39] Bilodeau B, Jaques N, Koh PW, Kim B. Impossibility theorems for feature attribution. Proc Natl Acad Sci. 2024;121(2): e2304406120. 38181057 10.1073/pnas.2304406120 PMC10786278

[40] Breitfuss A, Errou K, Kurteva A, Fensel A. Representing emotions with knowledge graphs for movie recommendations. Futur Gener Comput Syst. 2021;125:715–25.

[41] Zheng S, Rao J, Song Y, Zhang J, Xiao X, Fang EF, et al. PharmKG: a dedicated knowledge graph benchmark for biomedical data mining. Brief Bioinf. 2021;22(4):bbaa344. 10.1093/bib/bbaa344 33341877

[42] Chandak P, Huang K, Zitnik M. Building a knowledge graph to enable precision medicine. Sci Data. 2023;10(1):67. 36732524 10.1038/s41597-023-01960-3 PMC9893183

[43] Réda C.: TRANSCRIPT drug repurposing dataset. 10.5281/zenodo.7982976.Zenodo. Available from: 10.5281/zenodo.7982976.

[44] Yu HF, Bilenko M, Lin CJ. Selection of negative samples for one-class matrix factorization. In: Proceedings of the 2017 SIAM international conference on data mining. SIAM; 2017. p. 363–371.

[45] Spearman C. The proof and measurement of association between two things. Am J Psychol. 1987;100(3/4):441–71. 3322052

[46] Steyerberg E, Steyerberg E. Dealing with missing values. Clinical Prediction Models: A Practical Approach to Development, Validation, and Updating. 2009;p. 115–137.

[47] Le Morvan M, Josse J, Moreau T, Scornet E, Varoquaux G. NeuMiss networks: differentiable programming for supervised learning with missing values. Adv Neural Inf Process Syst. 2020;33:5980–90.

[48] Wang X, Ji H, Shi C, Wang B, Ye Y, Cui P, et al. Heterogeneous graph attention network. In: The world wide web conference; 2019. p. 2022–2032.

[49] Réda C.: PREDICT drug repurposing dataset. 10.5281/zenodo.7983090.Zenodo. Available from: 10.5281/zenodo.7983090.

[50] Luo H, Wang J, Li M, Luo J, Peng X, Wu FX, et al. Drug repositioning based on comprehensive similarity measures and bi-random walk algorithm. Bioinformatics. 2016;32(17):2664–71. 27153662 10.1093/bioinformatics/btw228

[51] Liang X, Zhang P, Yan L, Fu Y, Peng F, Qu L, et al. LRSSL: predict and interpret drug-disease associations based on data integration using sparse subspace learning. Bioinformatics. 2017;33(8):1187–96. 28096083 10.1093/bioinformatics/btw770

[52] Gao CQ, Zhou YK, Xin XH, Min H, Du PF. DDA-SKF: predicting drug-disease associations using similarity kernel fusion. Front Pharmacol. 2022;12: 784171. 35095495 10.3389/fphar.2021.784171 PMC8792612

[53] Ioannidis VN, Song X, Manchanda S, Li M, Pan X, Zheng D, et al.: DRKG—drug repurposing knowledge graph for Covid-19. https://github.com/gnn4dr/DRKG/.

[54] Himmelstein DS, Lizee A, Hessler C, Brueggeman L, Chen SL, Hadley D, et al. Systematic integration of biomedical knowledge prioritizes drugs for repurposing. Elife. 2017;6: e26726. 28936969 10.7554/eLife.26726 PMC5640425

[55] Ali M, Berrendorf M, Hoyt CT, Vermue L, Sharifzadeh S, Tresp V, et al. PyKEEN 1.0: a Python library for training and evaluating knowledge graph embeddings. J Mach Learn Res. 2021;22(82):1–6.

[56] Szklarczyk D, Kirsch R, Koutrouli M, Nastou K, Mehryary F, Hachilif R, et al. The STRING database in 2023: protein-protein association networks and functional enrichment analyses for any sequenced genome of interest. Nucleic Acids Res. 2023;51(D1):D638–46. 36370105 10.1093/nar/gkac1000 PMC9825434

[57] Pedregosa F, Varoquaux G, Gramfort A, Michel V, Thirion B, Grisel O, et al. Scikit-learn: machine learning in Python. J Mach Learn Res. 2011;12:2825–30.

[58] Imambi S, Prakash KB, Kanagachidambaresan G. PyTorch. Programming with TensorFlow: Solution for Edge Computing Applications; 2021. p. 87–104.

[59] Fey M, Lenssen JE. Fast graph representation learning with PyTorch Geometric. (preprint). 2019;arXiv preprint arXiv:1903.02428.

[60] Liberzon A, Birger C, Thorvaldsdóttir H, Ghandi M, Mesirov JP, Tamayo P. The molecular signatures database hallmark gene set collection. Cell Syst. 2015;1(6):417–25. 26771021 10.1016/j.cels.2015.12.004 PMC4707969

[61] Hubert L, Arabie P. Comparing partitions. J Class. 1985;2:193–218.

[62] Campello RJ, Moulavi D, Sander J. Density-based clustering based on hierarchical density estimates. In: Pacific-Asia conference on knowledge discovery and data mining. Springer; 2013. p. 160–172.

[63] Knox C, Wilson M, Klinger CM, Franklin M, Oler E, Wilson A, et al. DrugBank 6.0: the DrugBank knowledgebase for 2024. Nucleic Acids Res. 2024;52(D1):D1265–75. 37953279 10.1093/nar/gkad976 PMC10767804

[64] Kim S, Chen J, Cheng T, Gindulyte A, He J, He S, et al. PubChem 2023 update. Nucleic Acids Res. 2023;51(D1):D1373–80. 36305812 10.1093/nar/gkac956 PMC9825602

[65] Elizarraras JM, Liao Y, Shi Z, Zhu Q, Pico AR, Zhang B. WebGestalt 2024: faster gene set analysis and new support for metabolomics and multi-omics. Nucleic Acids Research. 2024;p. gkae456. 10.1093/nar/gkae456 PMC11223849 38808672

[66] Ha CE, Bhagavan N. Essentials of medical biochemistry: with clinical cases. Cambridge: Academic Press; 2011.

[67] Hubková B, Valko-Rokytovská M, Čižmárová B, Zábavníková M, Mareková M, Birková A. Tryptophan: its metabolism along the kynurenine, serotonin, and indole pathway in malignant melanoma. Int J Mol Sci. 2022;23(16):9160. 36012419 10.3390/ijms23169160 PMC9408957

[68] Oldan JD, Giglio BC, Smith E, Zhao W, Bouchard DM, Ivanovic M, et al. Increased tryptophan, but not increased glucose metabolism, predict resistance of pembrolizumab in stage III/IV melanoma. Oncoimmunology. 2023;12(1):2204753. 37123046 10.1080/2162402X.2023.2204753 PMC10142396

[69] Demopoulos HB. Effects of reducing the phenylalanine-Tyrosine intake of patients with advanced malignant melanoma. Cancer. 1966;19(5):657–64. 5930190 10.1002/1097-0142(196605)19:5<657::aid-cncr2820190509>3.0.co;2-j

[70] Lawson DH, Stockton LH, Bleier JC, Acosta PB, Heymsfield SB, Nixon DW. The effect of a phenylalanine and tyrosine restricted diet on elemental balance studies and plasma aminograms of patients with disseminated malignant melanoma. Am J Clin Nutr. 1985;41(1):73–84. 3966427 10.1093/ajcn/41.1.73

